# Supplementary material for: µPhos: a scalable and sensitive platform for high-dimensional phosphoproteomics
Source: Mol Syst Biol. 2024 Jun 21;20(8):8. doi: 10.1038/s44320-024-00050-9 (PMC11297287; doi:10.1038/s44320-024-00050-9)
Supplement: Supplementary file 1 — Appendix [file 44320_2024_50_MOESM1_ESM.pdf]

Appendix on

µPhos: a scalable and sensitive platform for high-dimensional phosphoproteomics

Table of Content

Appendix Figures

Appendix Figure S1	page 2
Appendix Figure S2	page 4
Appendix Figure S3	page 5
Appendix Figure S4	page 6

Appendix Tables

Appendix Table S1	page 7

Appendix Figures

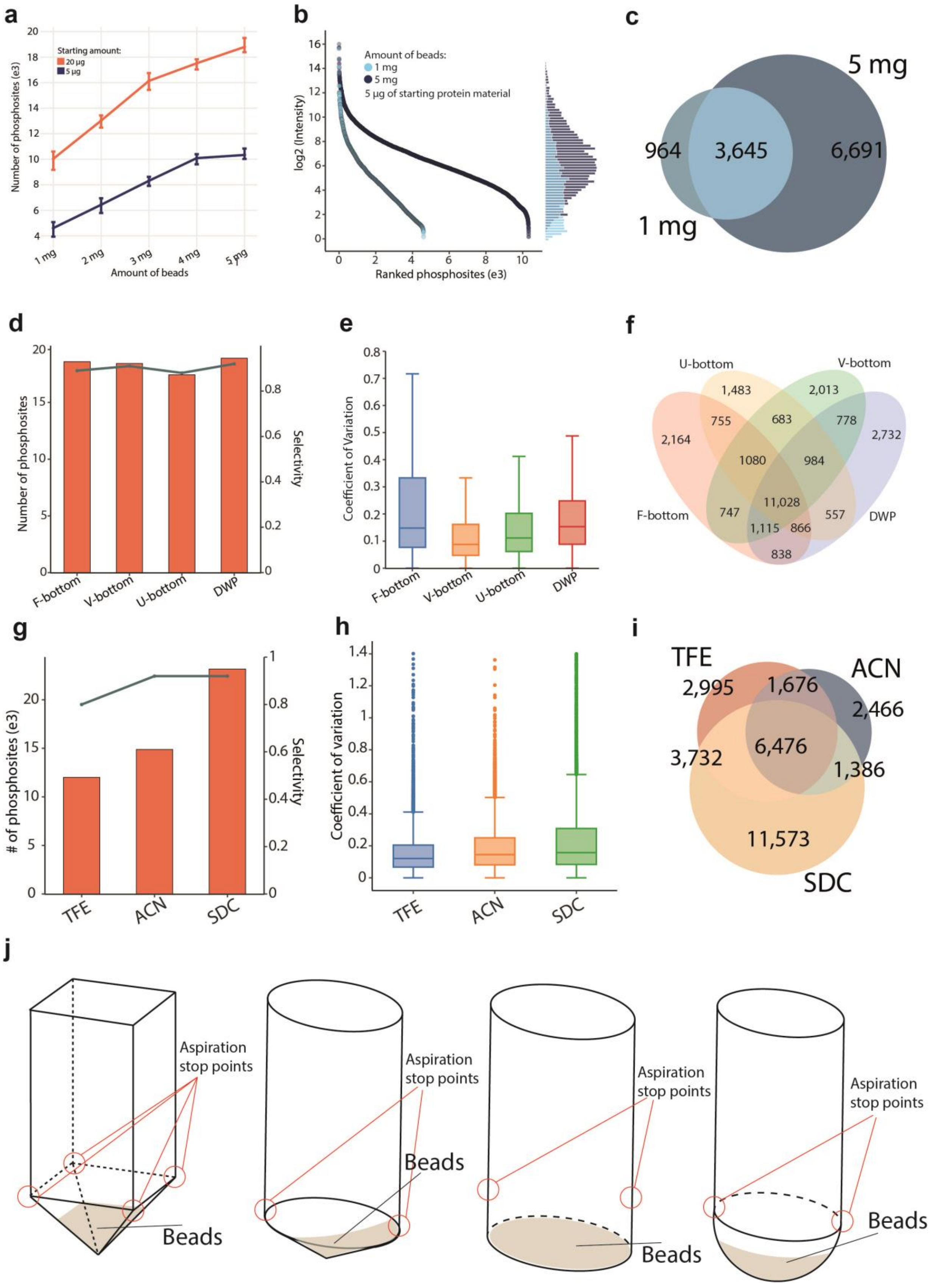

Appendix Figure S1. Parametrization of the µPhos protocol.

a Number of identified phosphopeptides from 5 and 20 µg a HeLa cell lysate as a function of TiO_2 bead amount.
b Ranked abundance of phosphosites from a.
c Overlap of identified phosphosites from a.
d Number of identified phosphopeptides from a HeLa cell lysate for different 96-well plate formats (flat-bottom, V-shaped bottom, U-shaped bottom and deep well plate).
e Coefficients of variation for the data in d.
f Overlap of phosphosites identified in d.
g Number of identified phosphopeptides from a HeLa cell lysate using different lysis buffers.
h Coefficients of variation for the data in g.
i Overlap of phosphosites identified in g.
j Recommended aspiration points for different well formats.

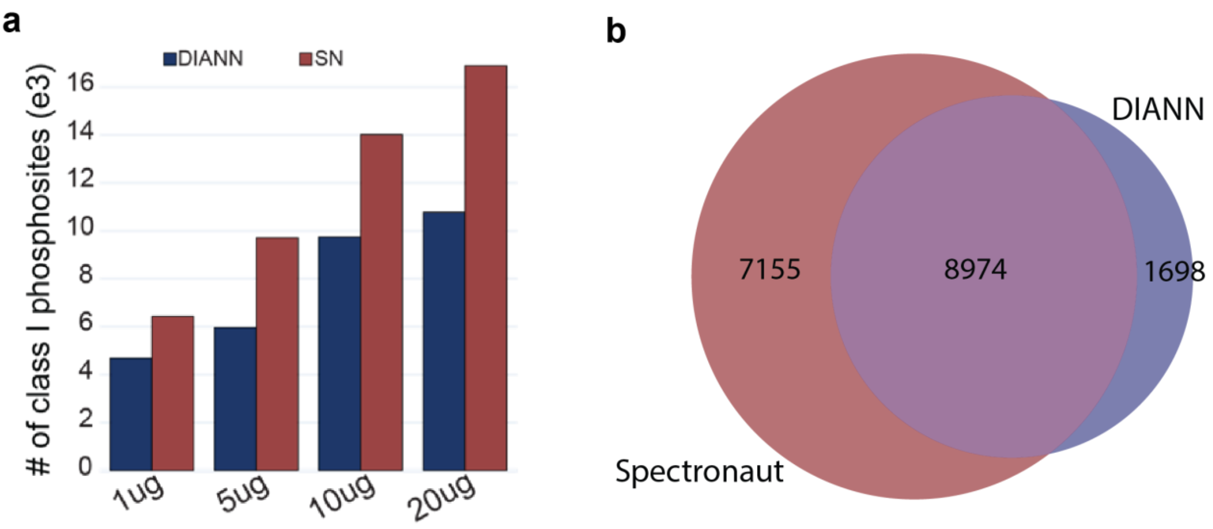

Appendix Figure S2. Comparison of different processing software for library-free data-independent experiments.
a Number of localized phosphosites (localization score >0.75) two alternative software packages (DIA-NN and Spectronaut (SN)) as a function of input amount of HeLa cell lysate.
b Overlap of base peptide sequences (i.e. regardless of modification and localization) identified by Spectronaut and DIA-NN from 20 µg input amount.

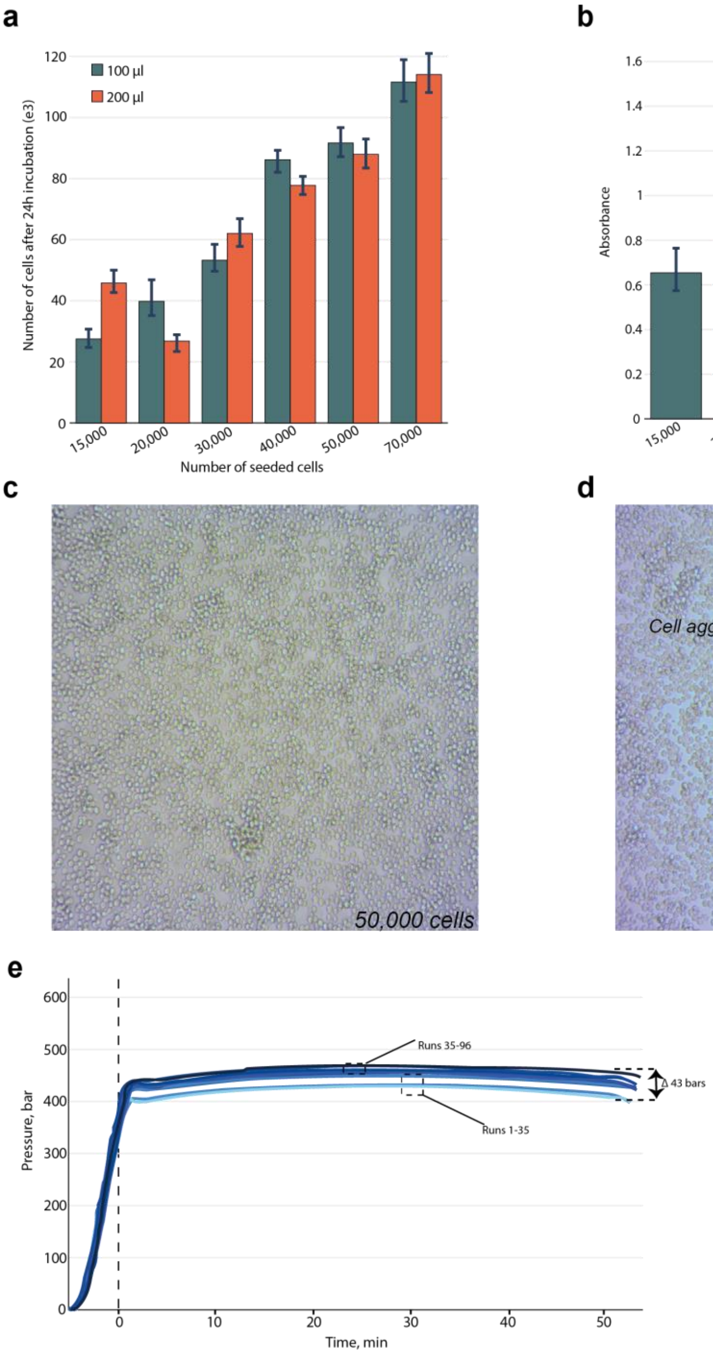

Appendix Figure S3. Application of µPhos to study tyrosine kinase inhibition in *BCR::ABL1* positive Ba/F3 cells.

a Cell count per well after 24 h for different numbers of seeded cells.

b Colorimetric cell viability assay.

c Representative image after 24 h of cells seeded at a density of 50,000 cells.

d Representative image after 24 h of cells seeded at a density of 70,000 cells.

e Liquid chromatography pressure profiles across the sequential measurement of 96 µPhos samples.

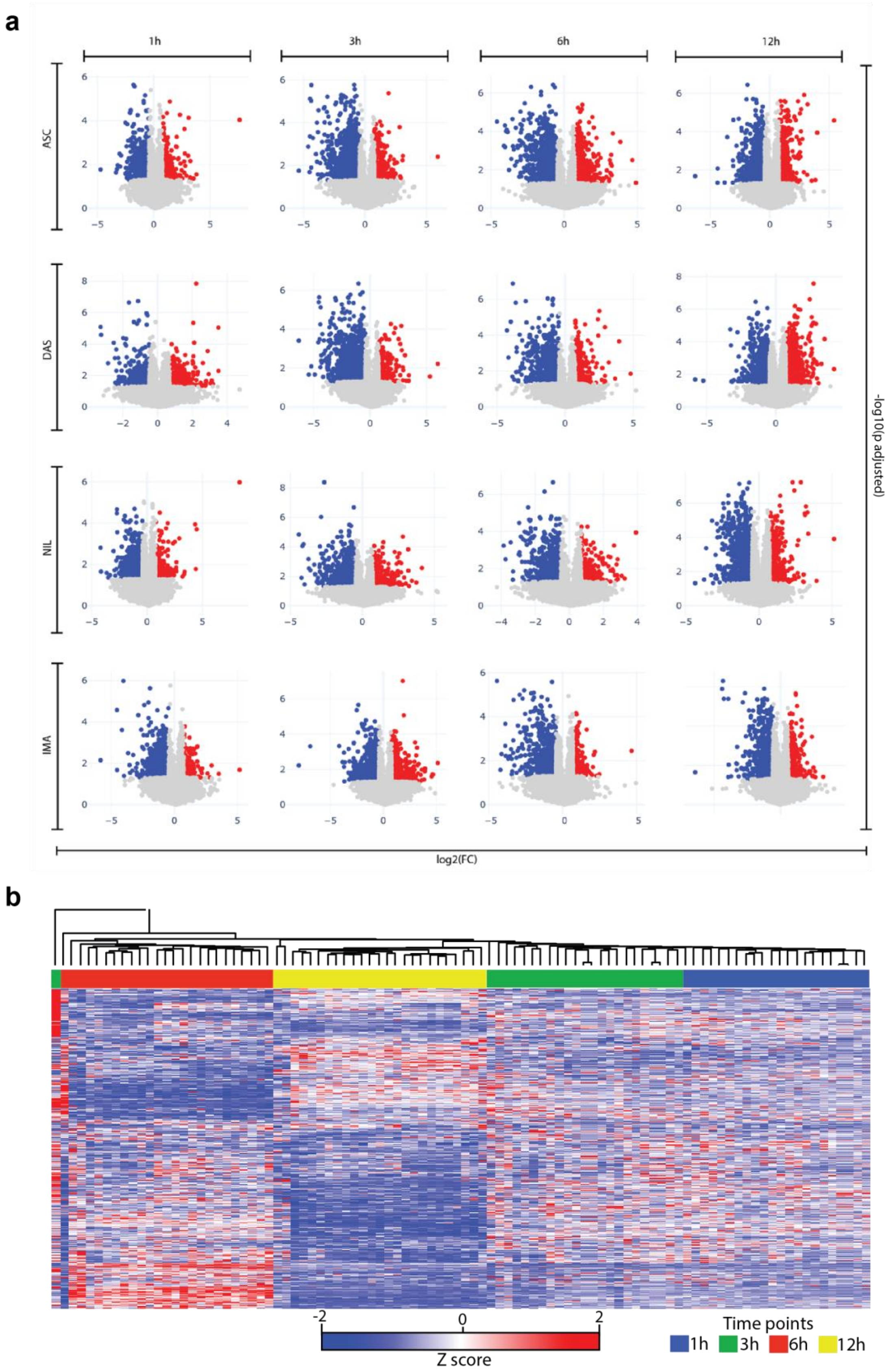

Appendix Figure S4. Statistical analysis of the tyrosine kinase inhibitor experiment.

a Pairwise volcano plots of all conditions (p adjusted < 0.05, absolute fold change > 1.5)

b Heatmap of ANOVA-significant phosphosites across all conditions (FDR < 0.01).

Appendix Tables

Appendix Table S1. Variable window size dia-PASEF method, optimized for phosphoproteome measurements.

#MS Type	Cycle Id	Start IM [1/K0]	End IM [1/K0]	Start Mass [m/z]	End Mass [m/z]
MS1	0	-	-	-	-
diaPASEF	1	0.92	1.41	847.29	872.84
diaPASEF	1	0.6	0.92	400.19	505.55
diaPASEF	2	0.95	1.41	872.84	898.63
diaPASEF	2	0.6	0.95	505.55	563.22
diaPASEF	3	0.97	1.41	898.63	924.44
diaPASEF	3	0.6	0.97	563.22	605.25
diaPASEF	4	0.99	1.41	924.44	950.77
diaPASEF	4	0.6	0.99	605.25	638.75
diaPASEF	5	1.01	1.41	950.77	977.9
diaPASEF	5	0.6	1.01	638.75	669.78
diaPASEF	6	1.03	1.41	977.9	1007.14
diaPASEF	6	0.6	1.03	669.78	699.82
diaPASEF	7	1.04	1.41	1007.14	1035.88
diaPASEF	7	0.6	1.04	699.82	727.79
diaPASEF	8	1.06	1.41	1035.88	1071.37
diaPASEF	8	0.6	1.06	727.79	752.97
diaPASEF	9	1.08	1.41	1071.37	1114.82
diaPASEF	9	0.6	1.08	752.97	775.32
diaPASEF	10	1.1	1.41	1114.82	1169.53
diaPASEF	10	0.6	1.1	775.32	799.99
diaPASEF	11	1.13	1.41	1169.53	1246.22
diaPASEF	11	0.6	1.13	799.99	824.35
diaPASEF	12	1.18	1.41	1246.22	1399.28
diaPASEF	12	0.6	1.18	824.35	847.29
