# Supplementary figures and images for: µPhos: a scalable and sensitive platform for high-dimensional phosphoproteomics
Source: Mol Syst Biol. 2024 Jun 21;20(8):8. doi: 10.1038/s44320-024-00050-9 (PMC11297287; doi:10.1038/s44320-024-00050-9)

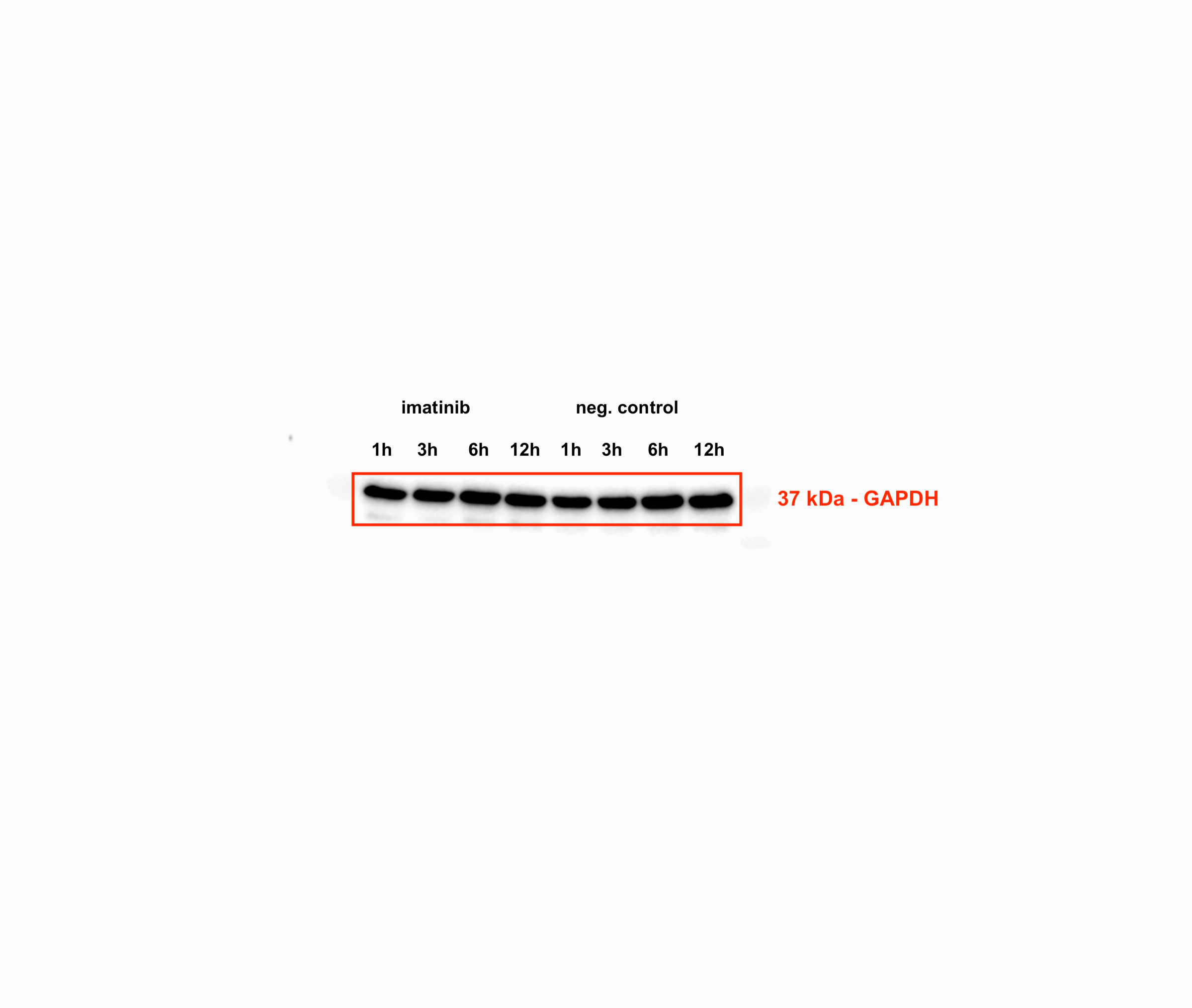

Supplement: Supplementary file 3 — Source data Fig. 5 [file 44320_2024_50_MOESM3_ESM.zip › Figure 5/5E/Crkl/WB_13_231124_Imatinib_02_GAPDH_3.tif]

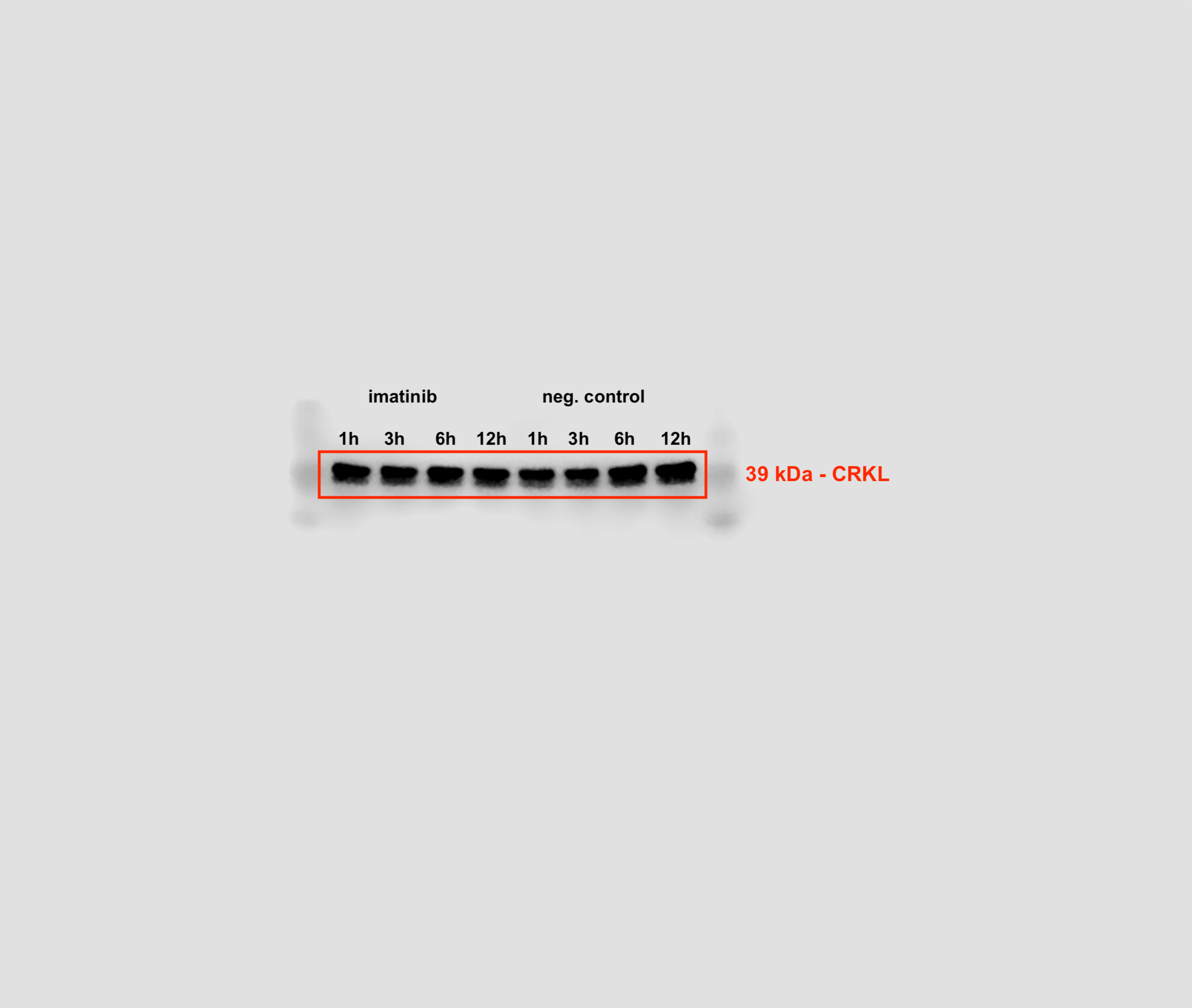

Supplement: Supplementary file 3 — Source data Fig. 5 [file 44320_2024_50_MOESM3_ESM.zip › Figure 5/5E/Crkl/WB_13_231127_Imatinib_02_CRKL_3.tif]

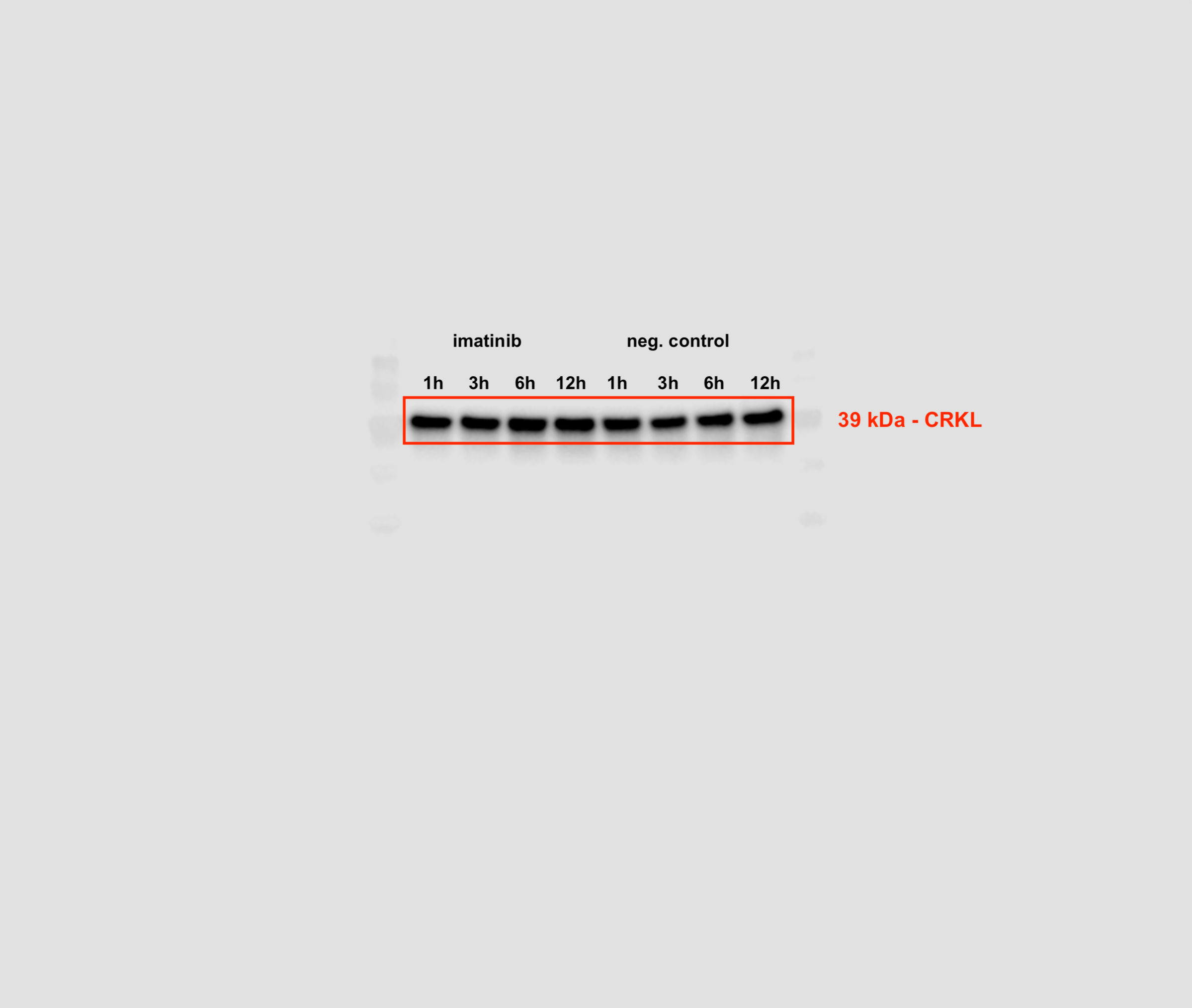

Supplement: Supplementary file 3 — Source data Fig. 5 [file 44320_2024_50_MOESM3_ESM.zip › Figure 5/5E/Crkl/WB_15_231129_02_CRKL_BaF3_BCRABL1_1.tif]

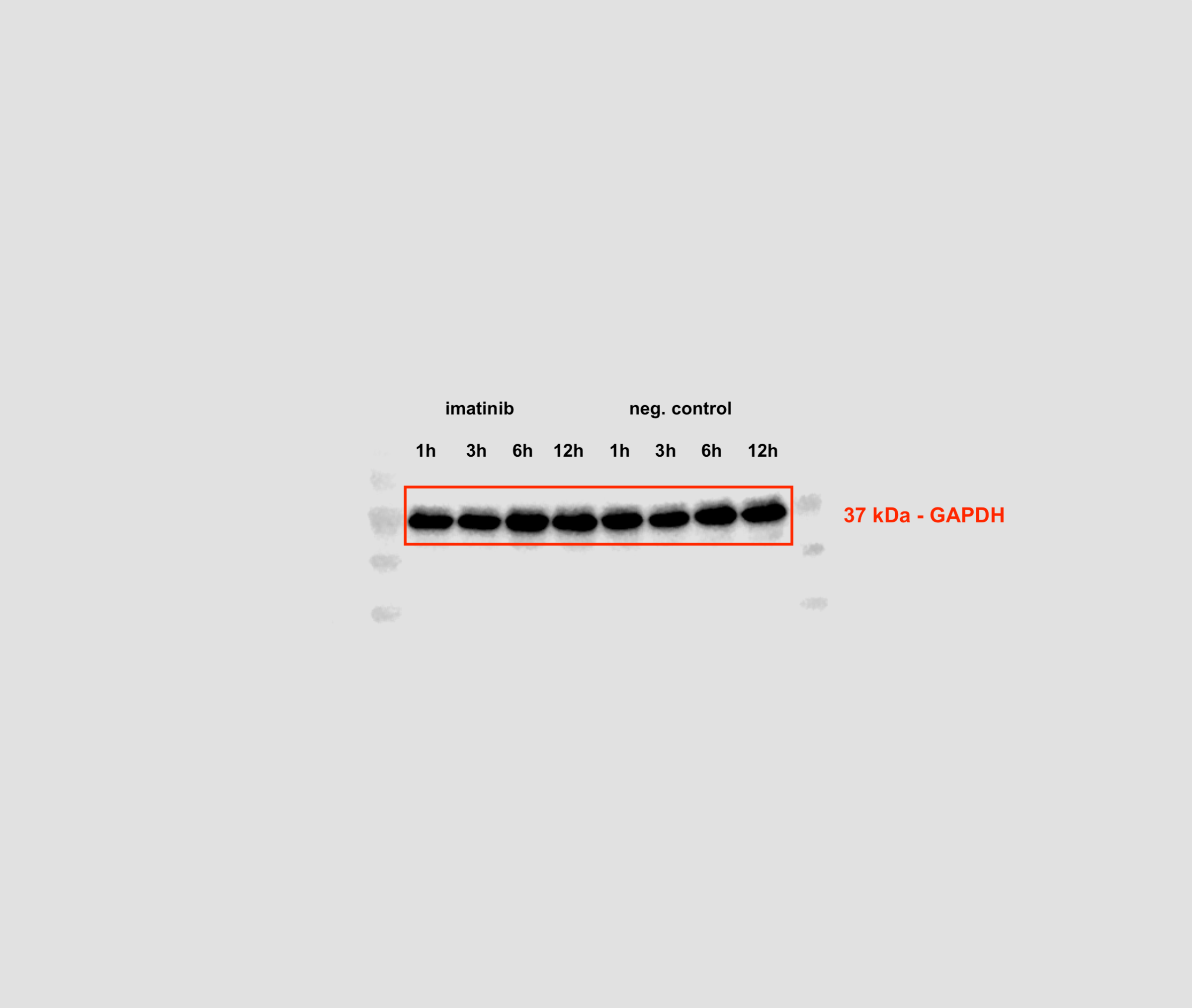

Supplement: Supplementary file 3 — Source data Fig. 5 [file 44320_2024_50_MOESM3_ESM.zip › Figure 5/5E/Crkl/WB_15_231130_02_GAPDH_BaF3_BCRABL1_1.tif]

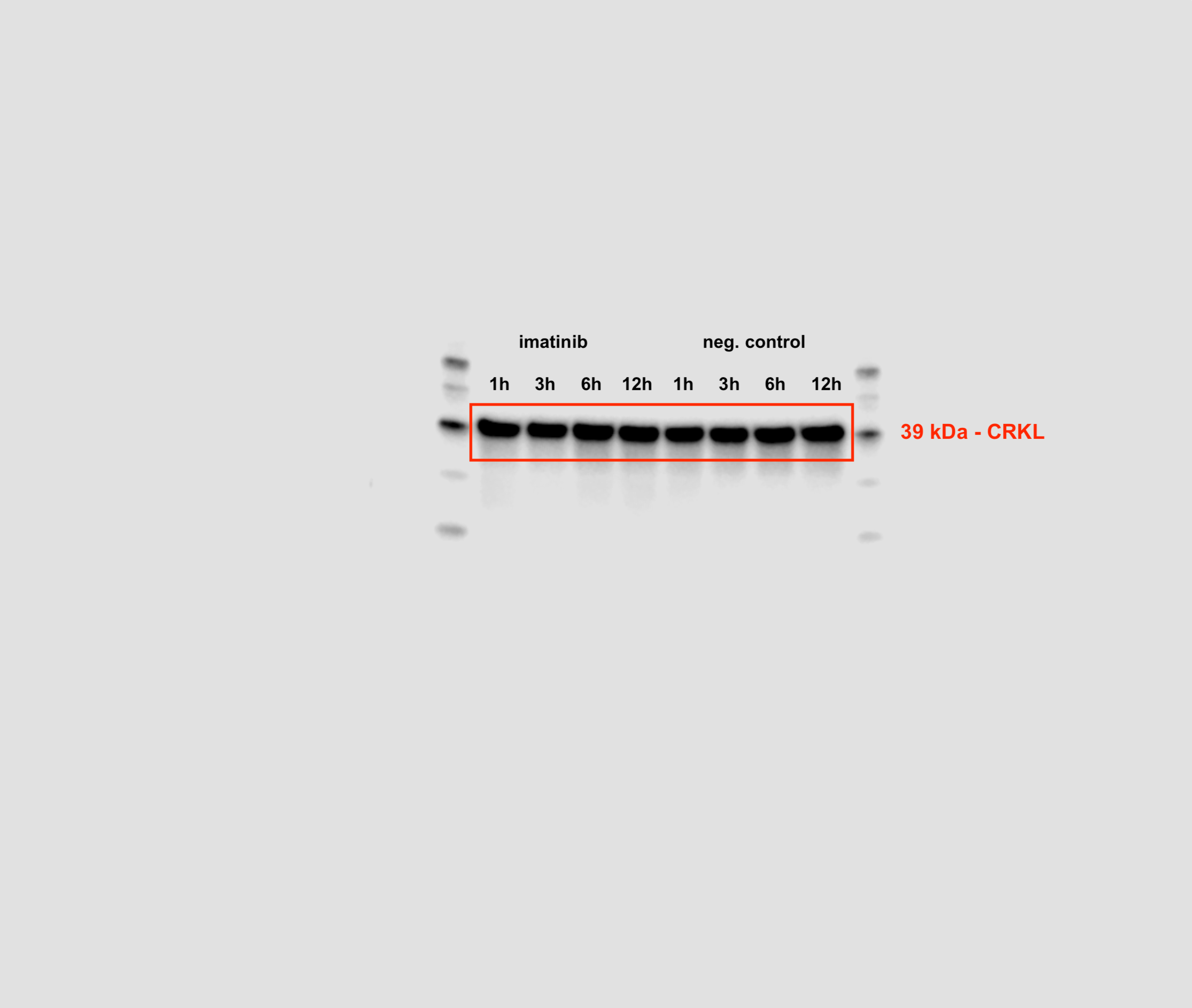

Supplement: Supplementary file 3 — Source data Fig. 5 [file 44320_2024_50_MOESM3_ESM.zip › Figure 5/5E/Crkl/WB_17_231130_01_CRKL_BaF3_BCRABL1_1.tif]

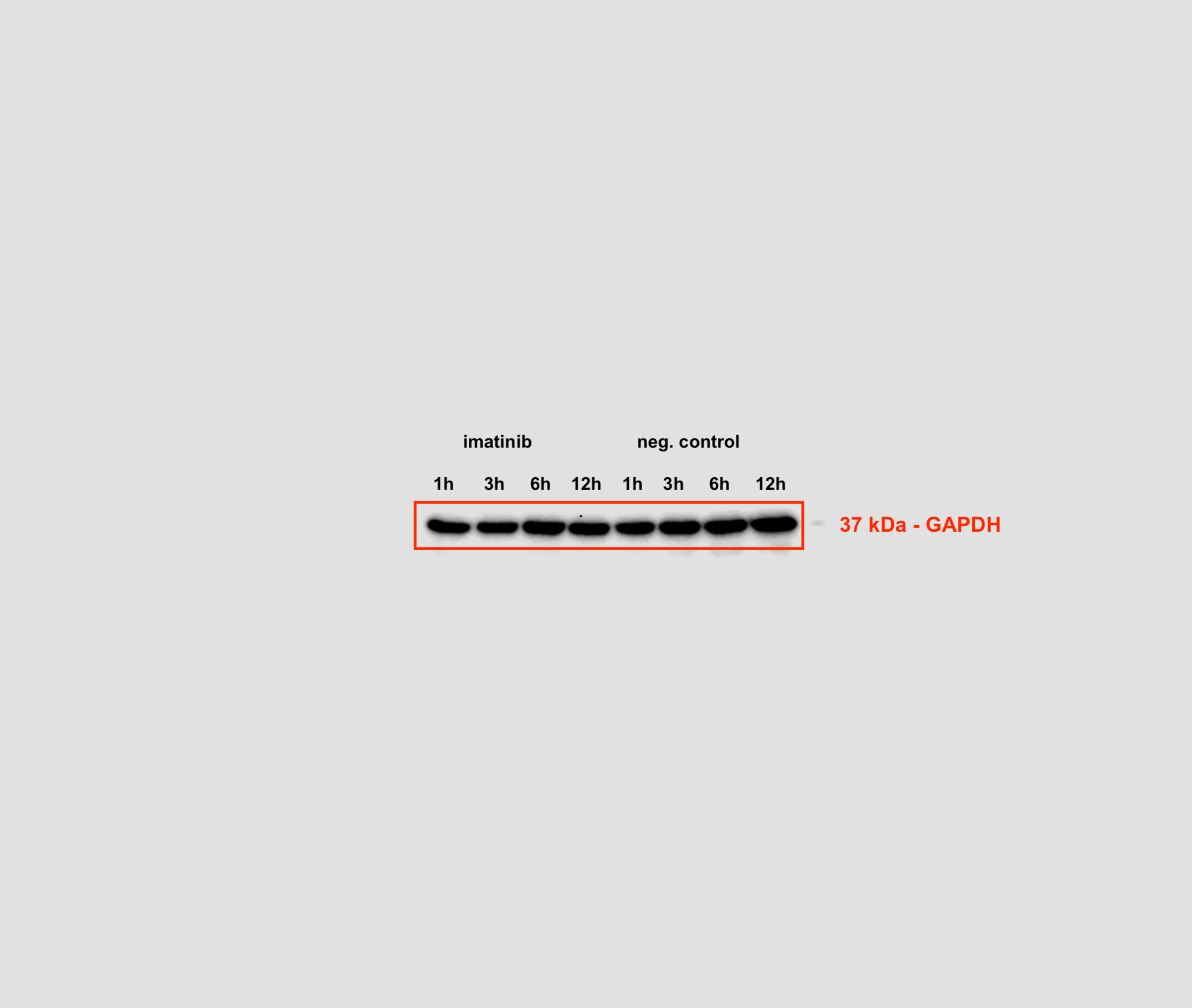

Supplement: Supplementary file 3 — Source data Fig. 5 [file 44320_2024_50_MOESM3_ESM.zip › Figure 5/5E/Crkl/WB_17_231201_01_GAPDH_BaF3_BCRABL1_1.tif]

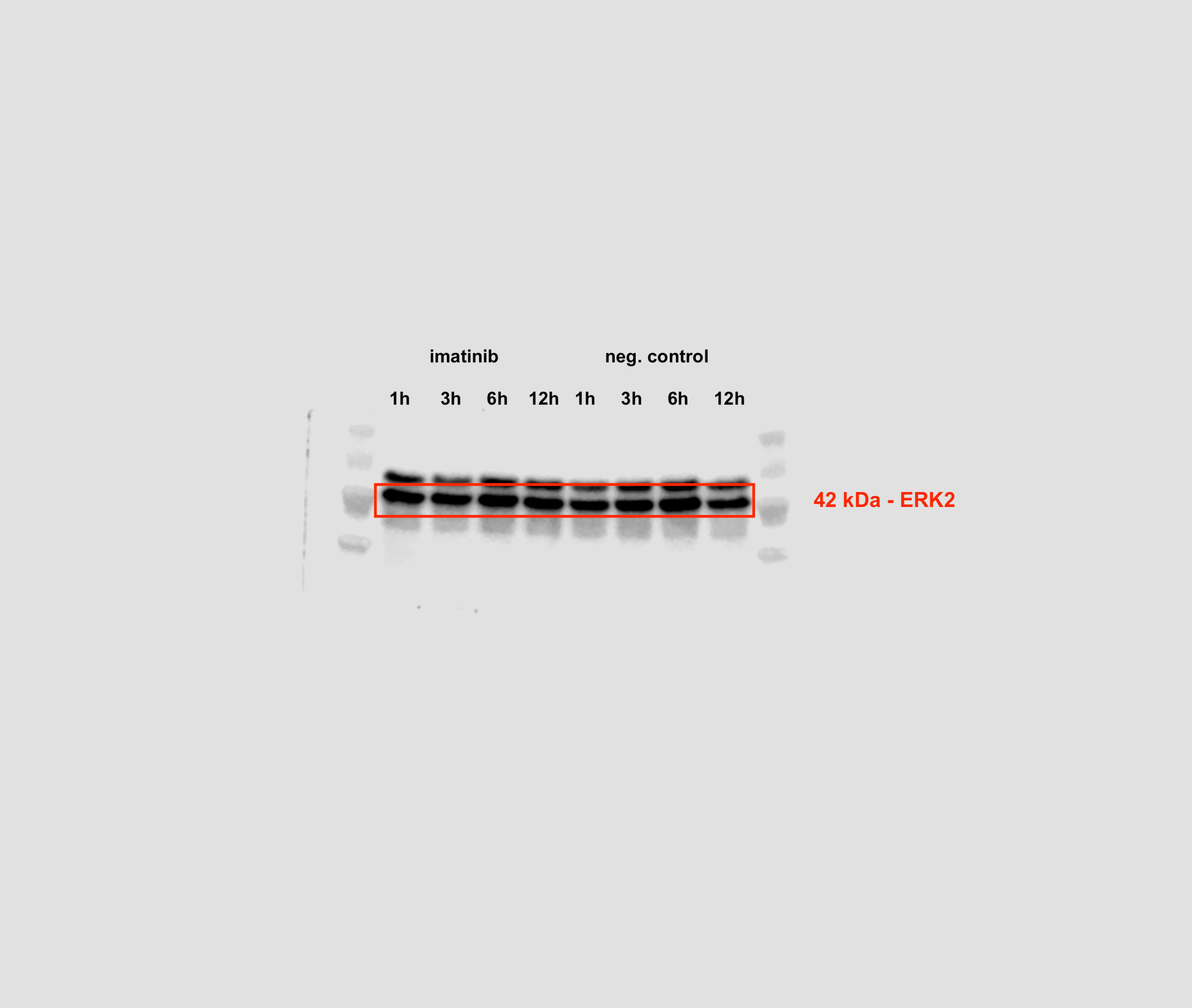

Supplement: Supplementary file 3 — Source data Fig. 5 [file 44320_2024_50_MOESM3_ESM.zip › Figure 5/5E/Erk/WB_13_231123_Imatinib_02_p44_p42_2.tif]

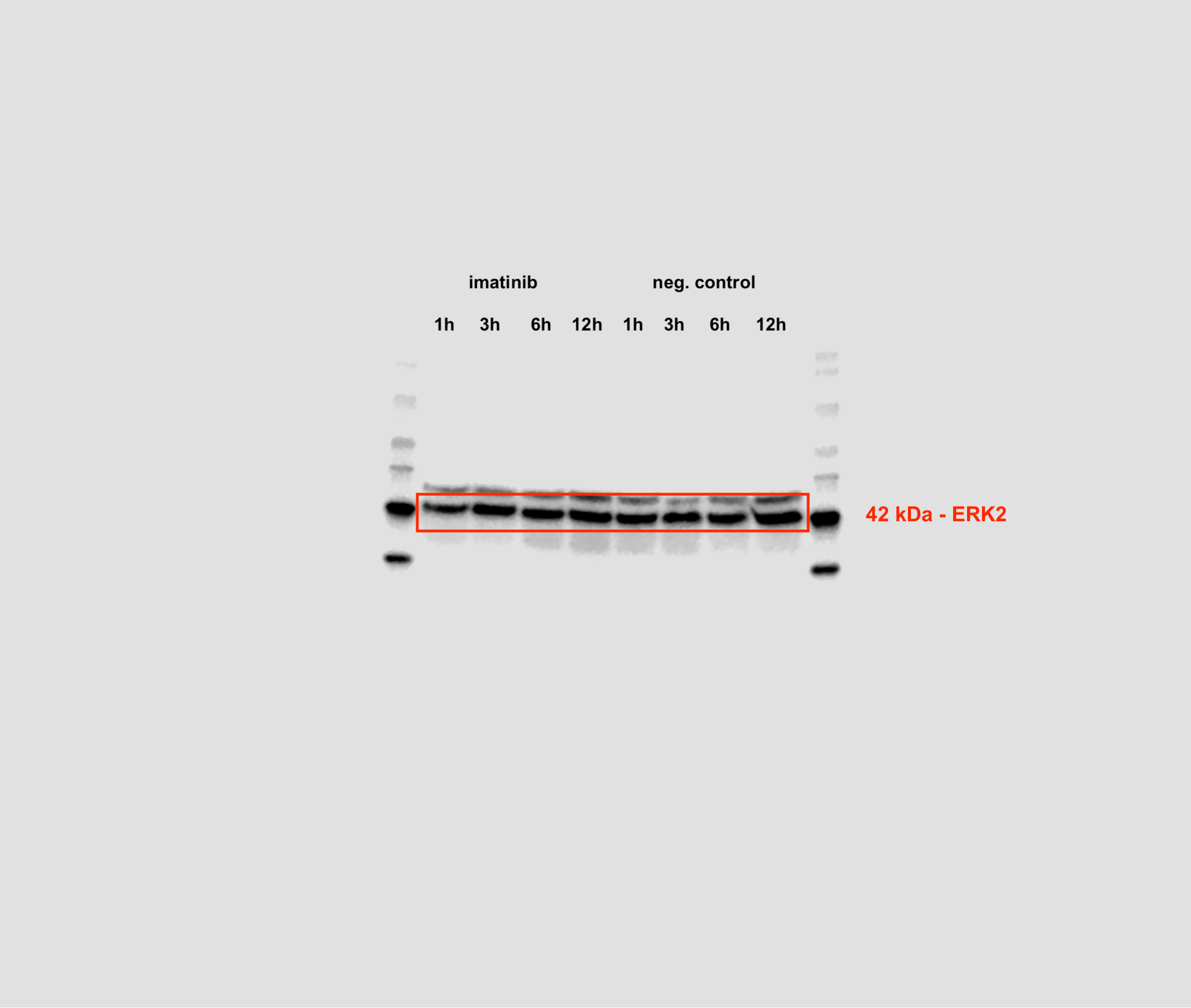

Supplement: Supplementary file 3 — Source data Fig. 5 [file 44320_2024_50_MOESM3_ESM.zip › Figure 5/5E/Erk/WB_22_231213_ERK1-2_BaF3-BCRABL1_Imatinib_3.tif]

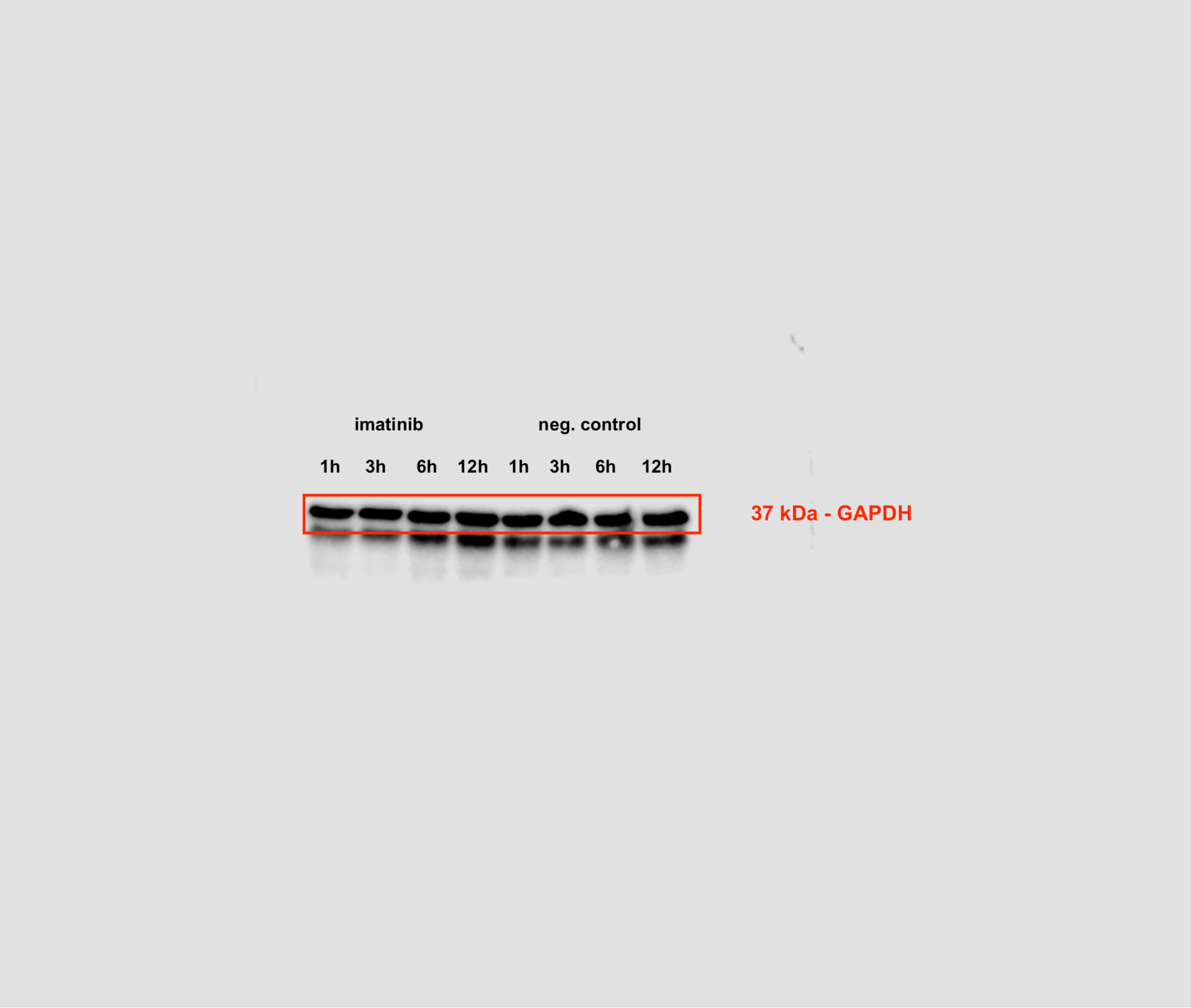

Supplement: Supplementary file 3 — Source data Fig. 5 [file 44320_2024_50_MOESM3_ESM.zip › Figure 5/5E/Erk/WB_22_231215_GAPDH_BaF3-BCRABL1_Imatinib_1.tif]

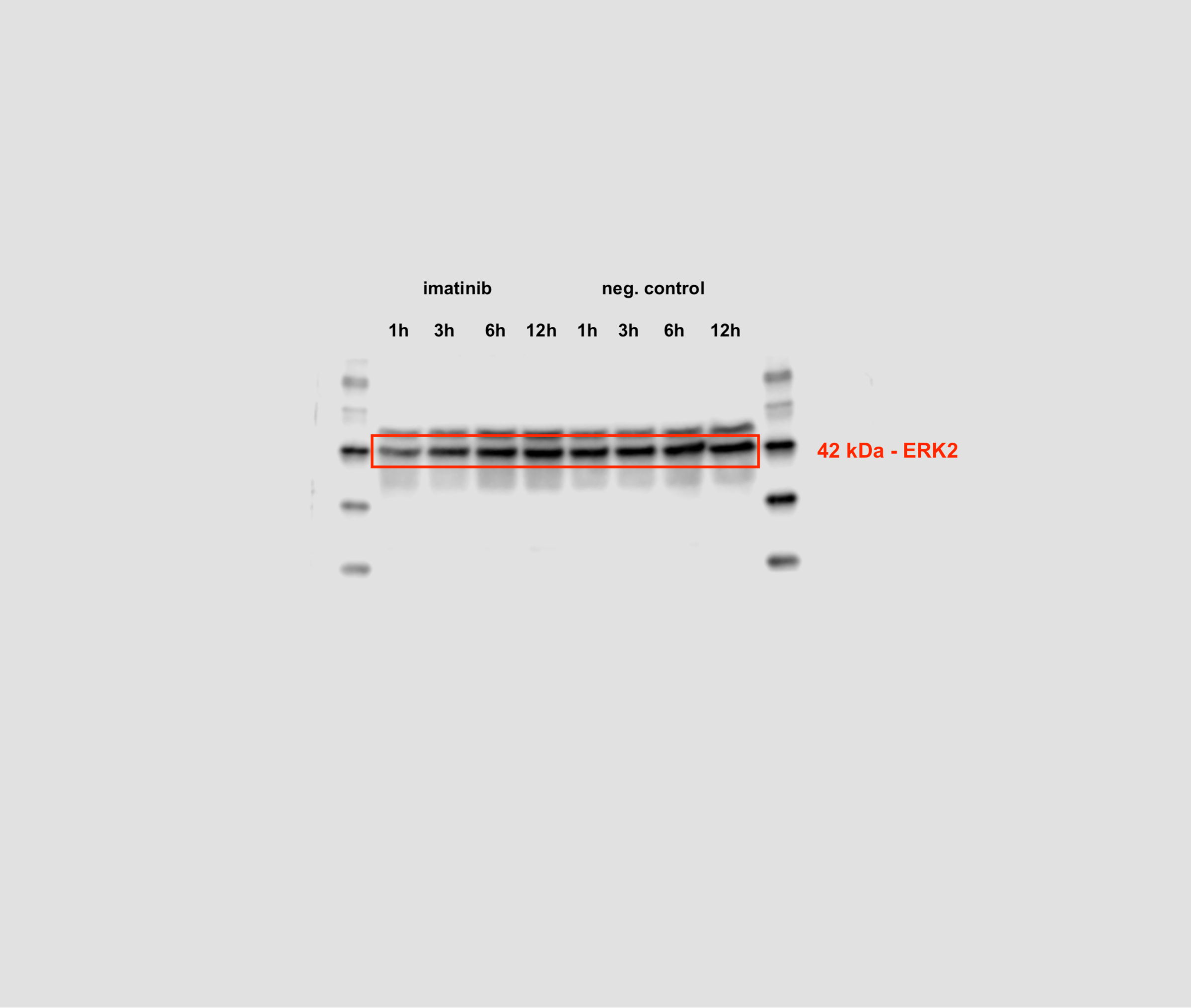

Supplement: Supplementary file 3 — Source data Fig. 5 [file 44320_2024_50_MOESM3_ESM.zip › Figure 5/5E/Erk/WB_23_231214_ERK1-2_BaF3-BCRABL1_Imatinib_2.tif]

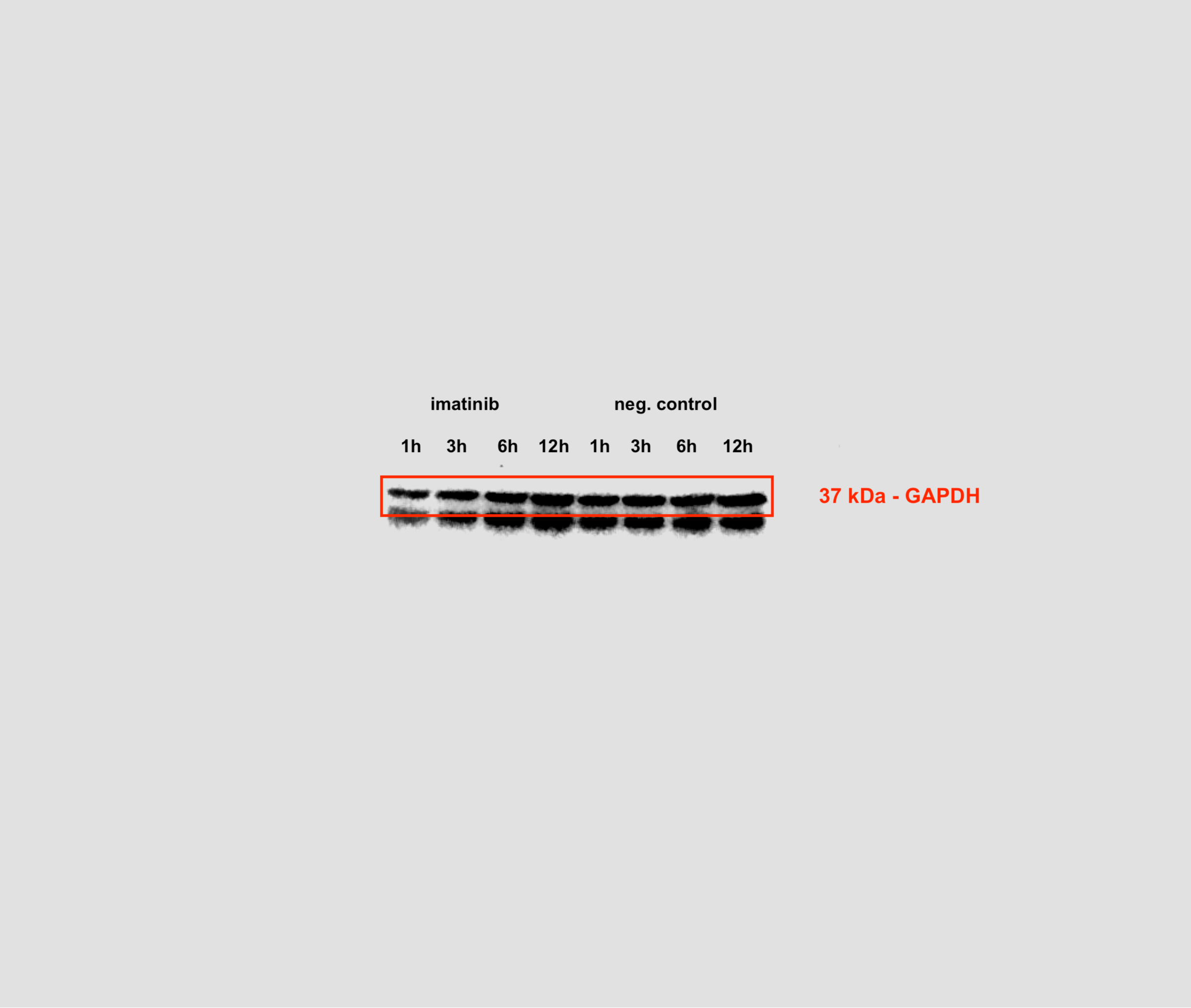

Supplement: Supplementary file 3 — Source data Fig. 5 [file 44320_2024_50_MOESM3_ESM.zip › Figure 5/5E/Erk/WB_23_231215_GAPDH_BaF3-BCRABL1_Imatinib_1.tif]

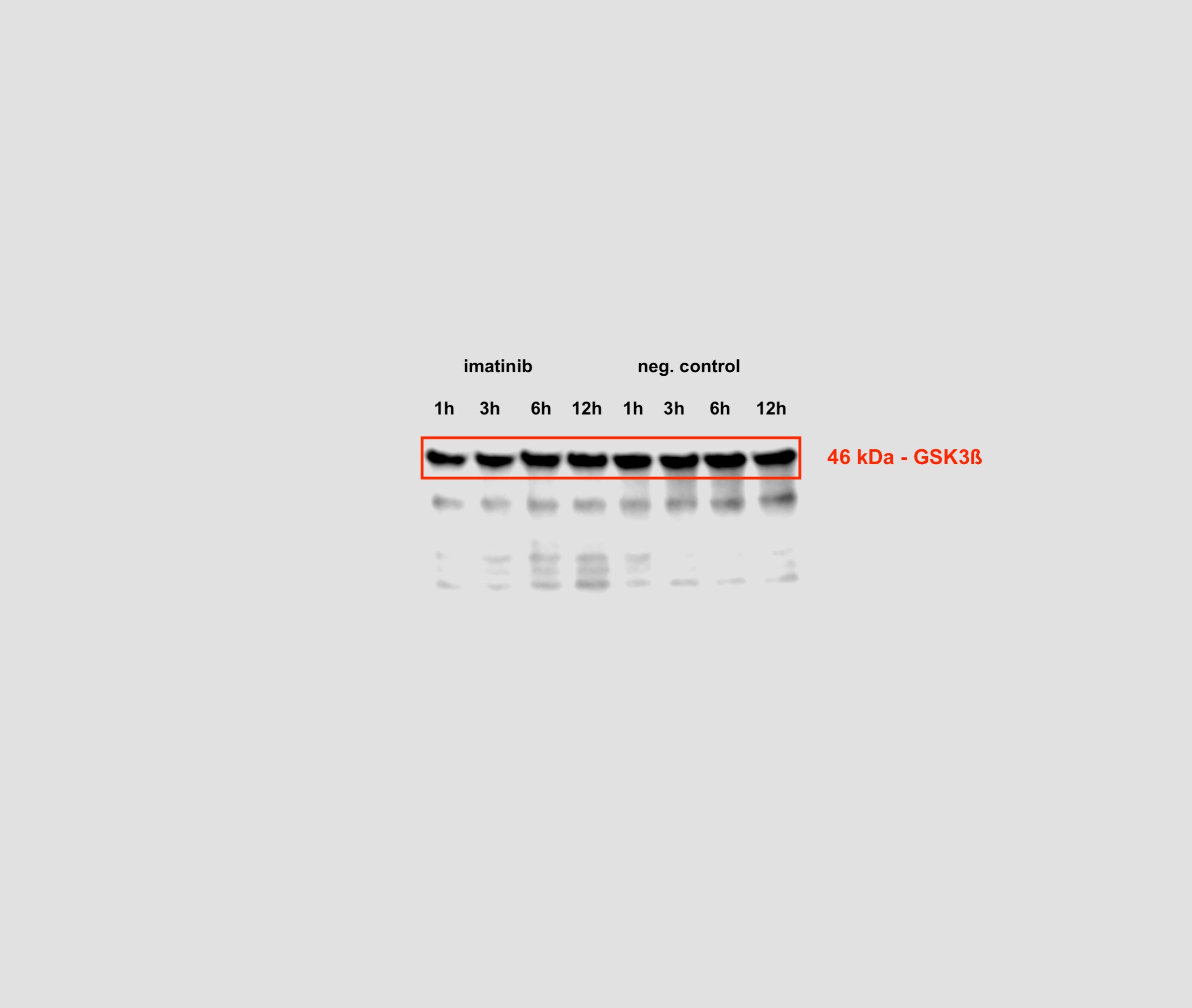

Supplement: Supplementary file 3 — Source data Fig. 5 [file 44320_2024_50_MOESM3_ESM.zip › Figure 5/5E/Gsk3b/WB_13_231123_Imatinib_01_GSK3b_2.tif]

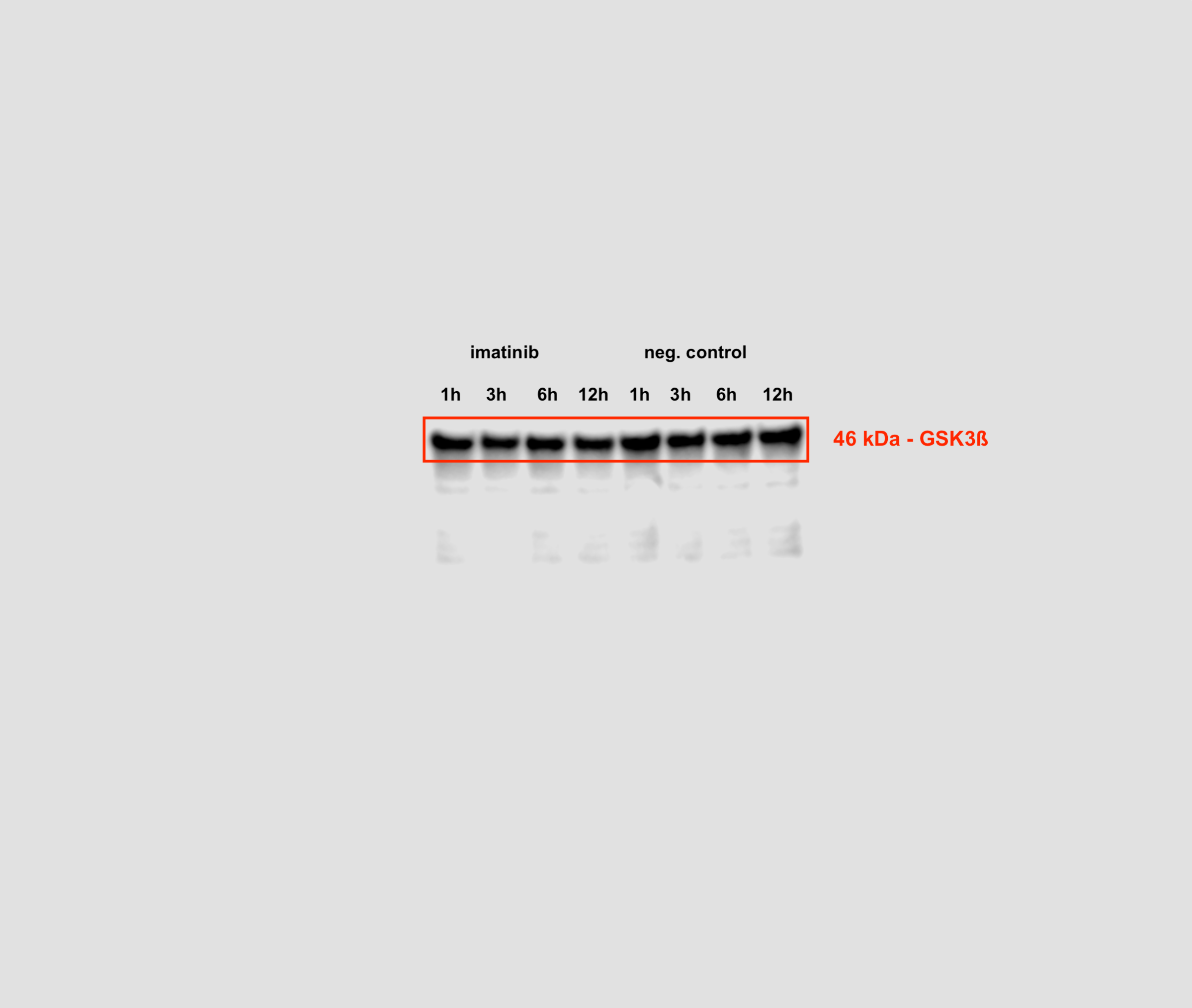

Supplement: Supplementary file 3 — Source data Fig. 5 [file 44320_2024_50_MOESM3_ESM.zip › Figure 5/5E/Gsk3b/WB_15_231201_01_GSK3b_BaF3_BCRABL1_3.tif]

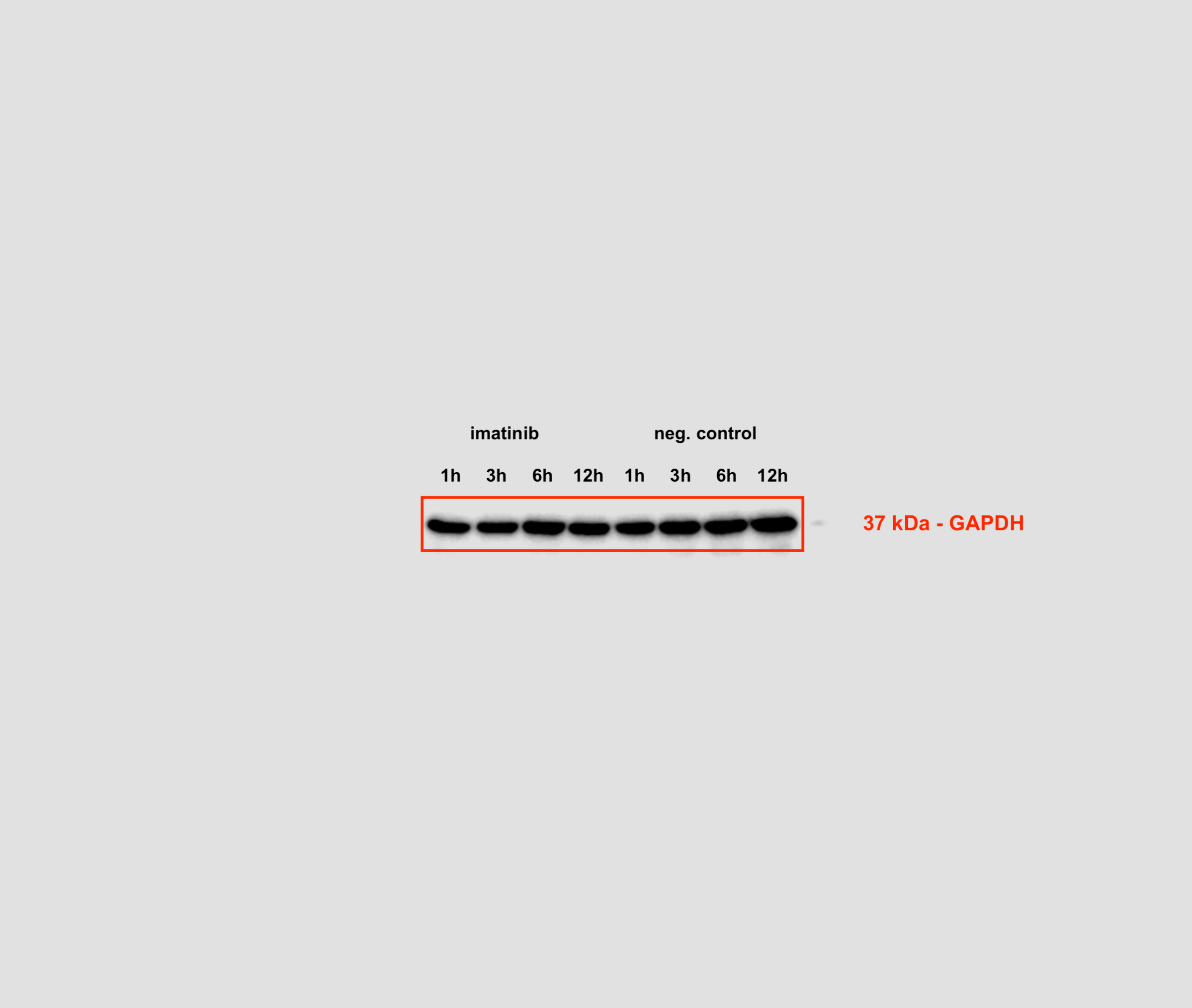

Supplement: Supplementary file 3 — Source data Fig. 5 [file 44320_2024_50_MOESM3_ESM.zip › Figure 5/5E/Gsk3b/WB_17_231201_01_GAPDH_BaF3_BCRABL1_1.tif]

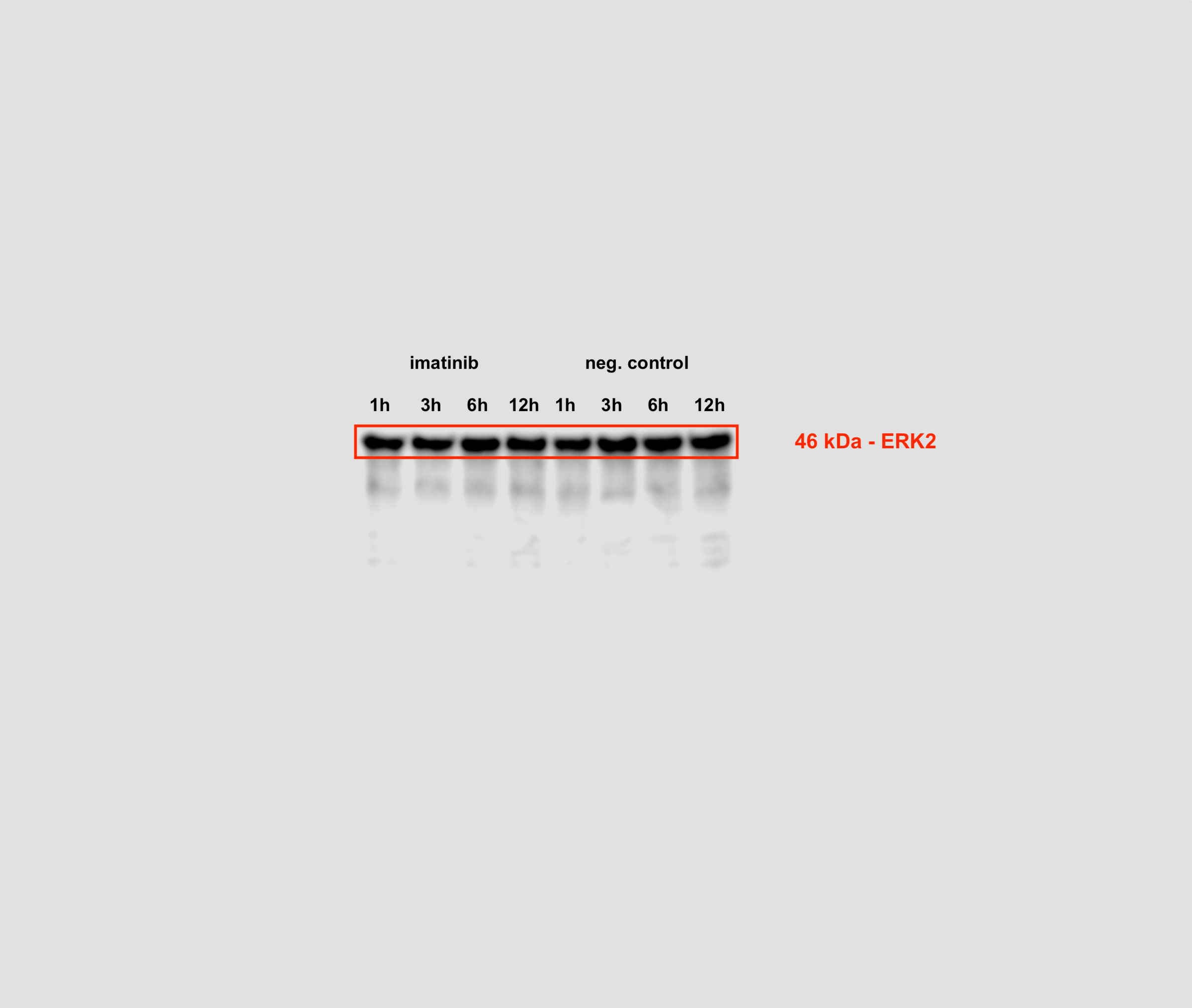

Supplement: Supplementary file 3 — Source data Fig. 5 [file 44320_2024_50_MOESM3_ESM.zip › Figure 5/5E/Gsk3b/WB_17_231201_02_GSK3b_BaF3_BCRABL1_2.tif]

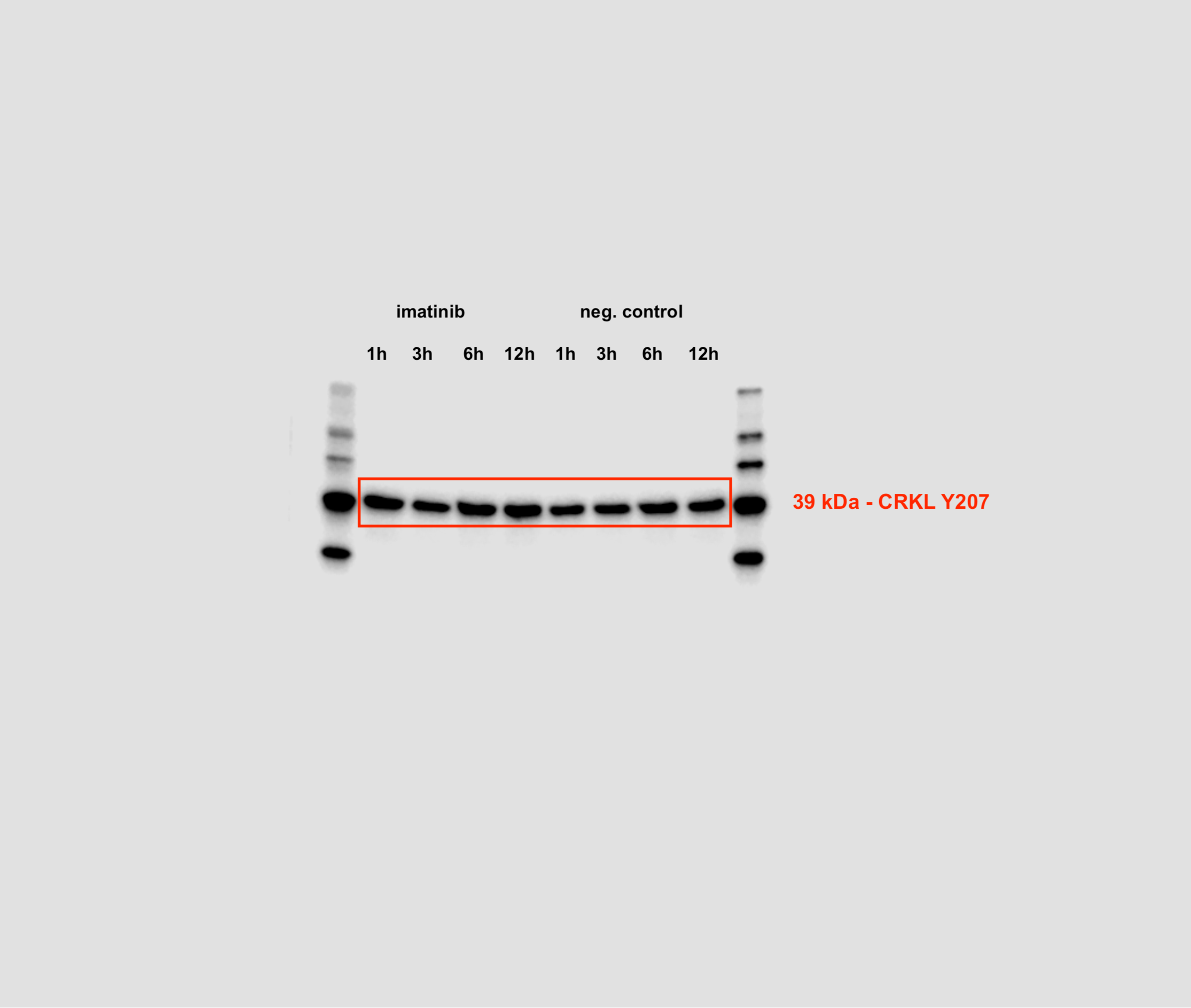

Supplement: Supplementary file 3 — Source data Fig. 5 [file 44320_2024_50_MOESM3_ESM.zip › Figure 5/5E/pCrkl/240125_pCRKL_Y207_Imatinib_05.tif]

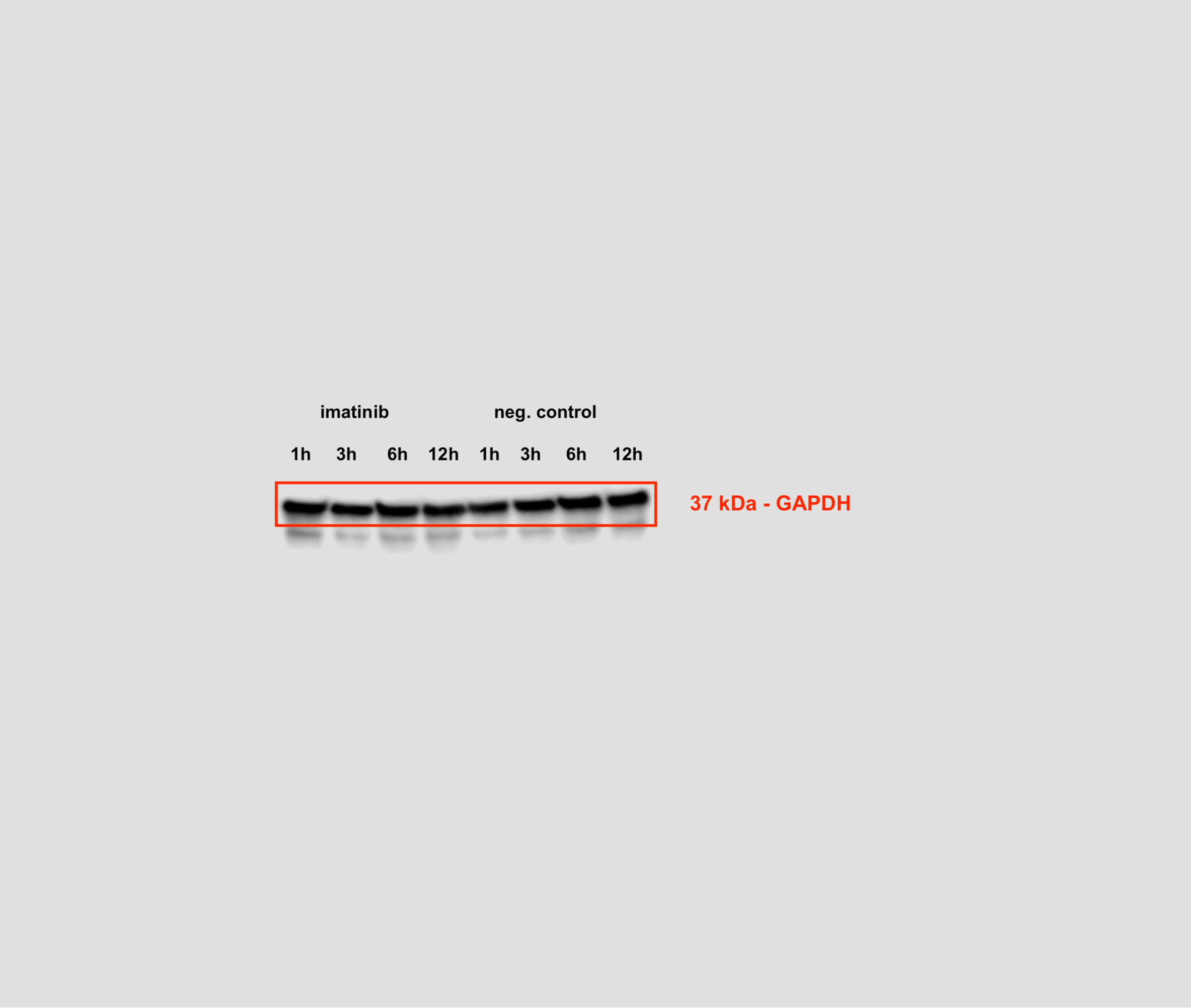

Supplement: Supplementary file 3 — Source data Fig. 5 [file 44320_2024_50_MOESM3_ESM.zip › Figure 5/5E/pCrkl/240126_GAPDH_Imatinib_4.tif]

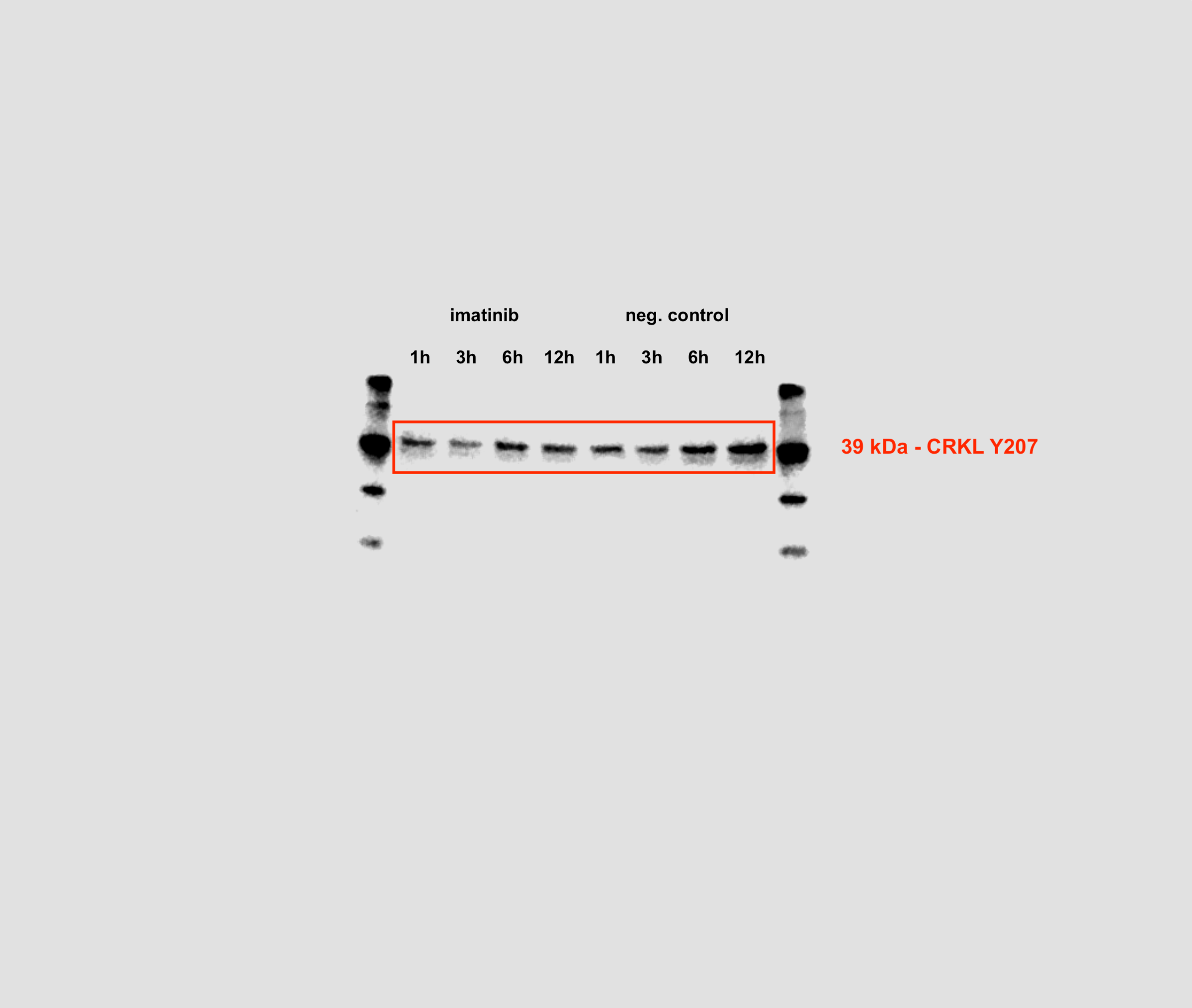

Supplement: Supplementary file 3 — Source data Fig. 5 [file 44320_2024_50_MOESM3_ESM.zip › Figure 5/5E/pCrkl/WB_15_231129_01_pCRKL_y207_BaF3_BCRABL1_3.tif]

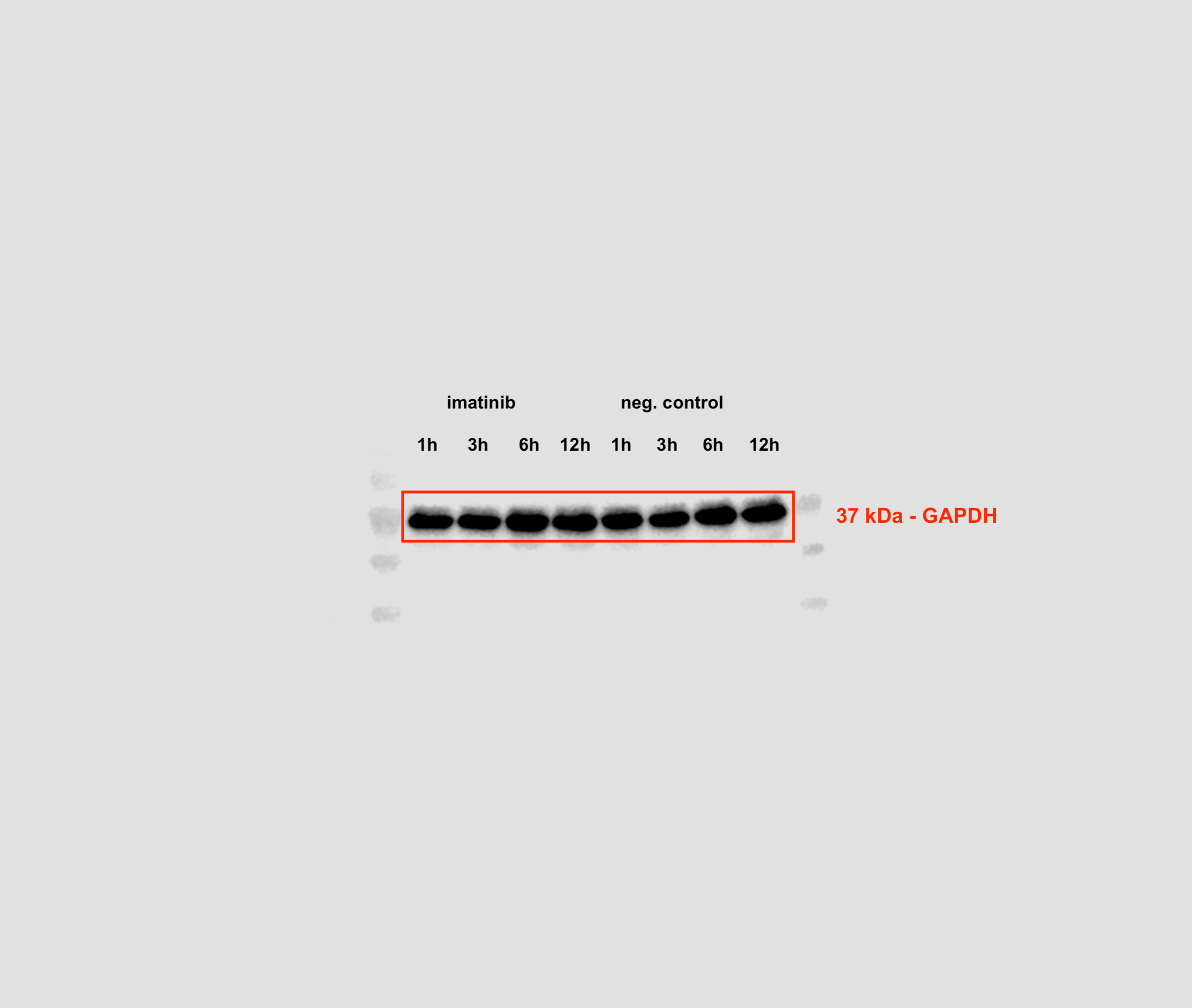

Supplement: Supplementary file 3 — Source data Fig. 5 [file 44320_2024_50_MOESM3_ESM.zip › Figure 5/5E/pCrkl/WB_15_231130_02_GAPDH_BaF3_BCRABL1_1.tif]

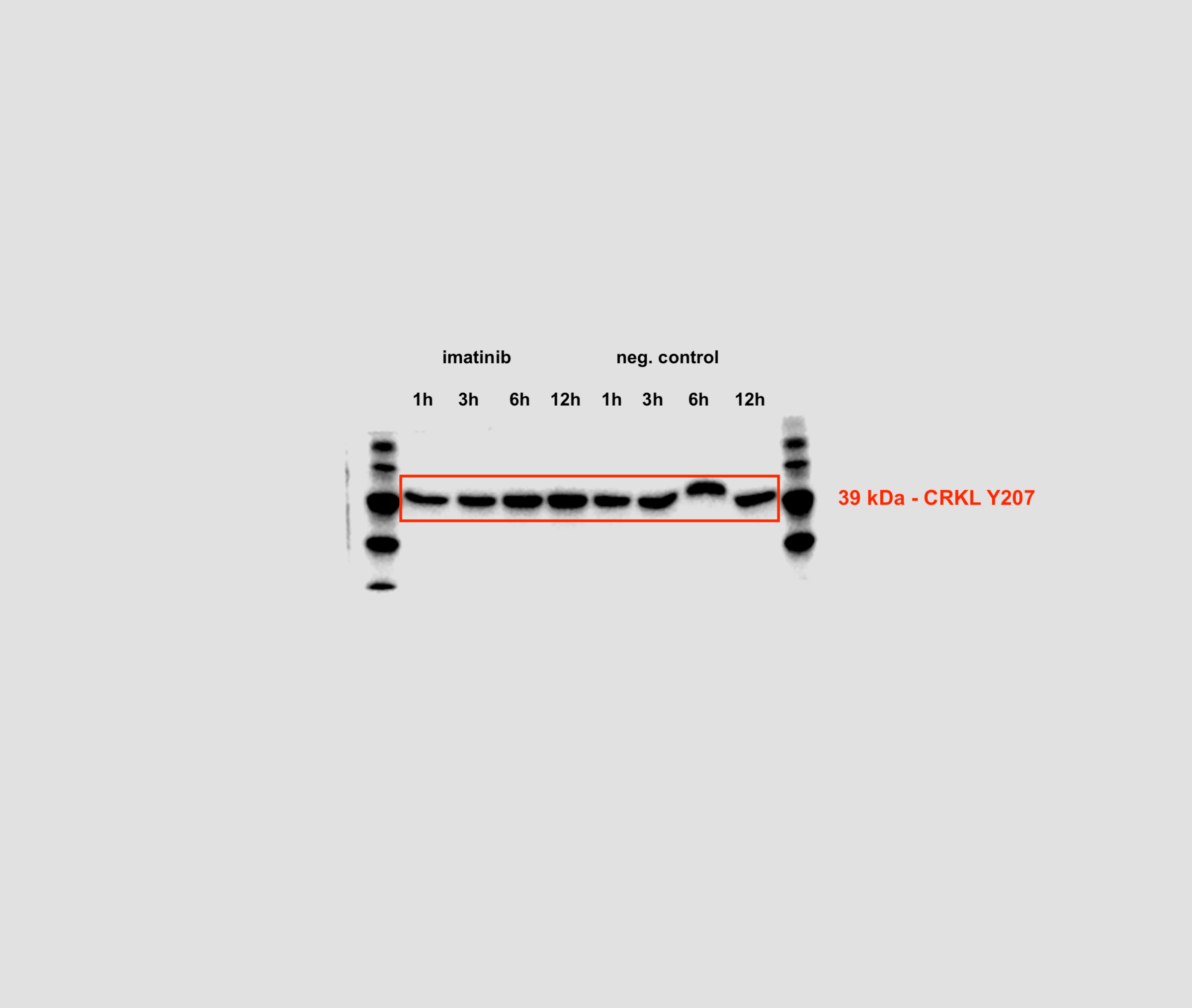

Supplement: Supplementary file 3 — Source data Fig. 5 [file 44320_2024_50_MOESM3_ESM.zip › Figure 5/5E/pCrkl/WB_19_231205_pCRKL_BaF3_BCRABL1_1.tif]

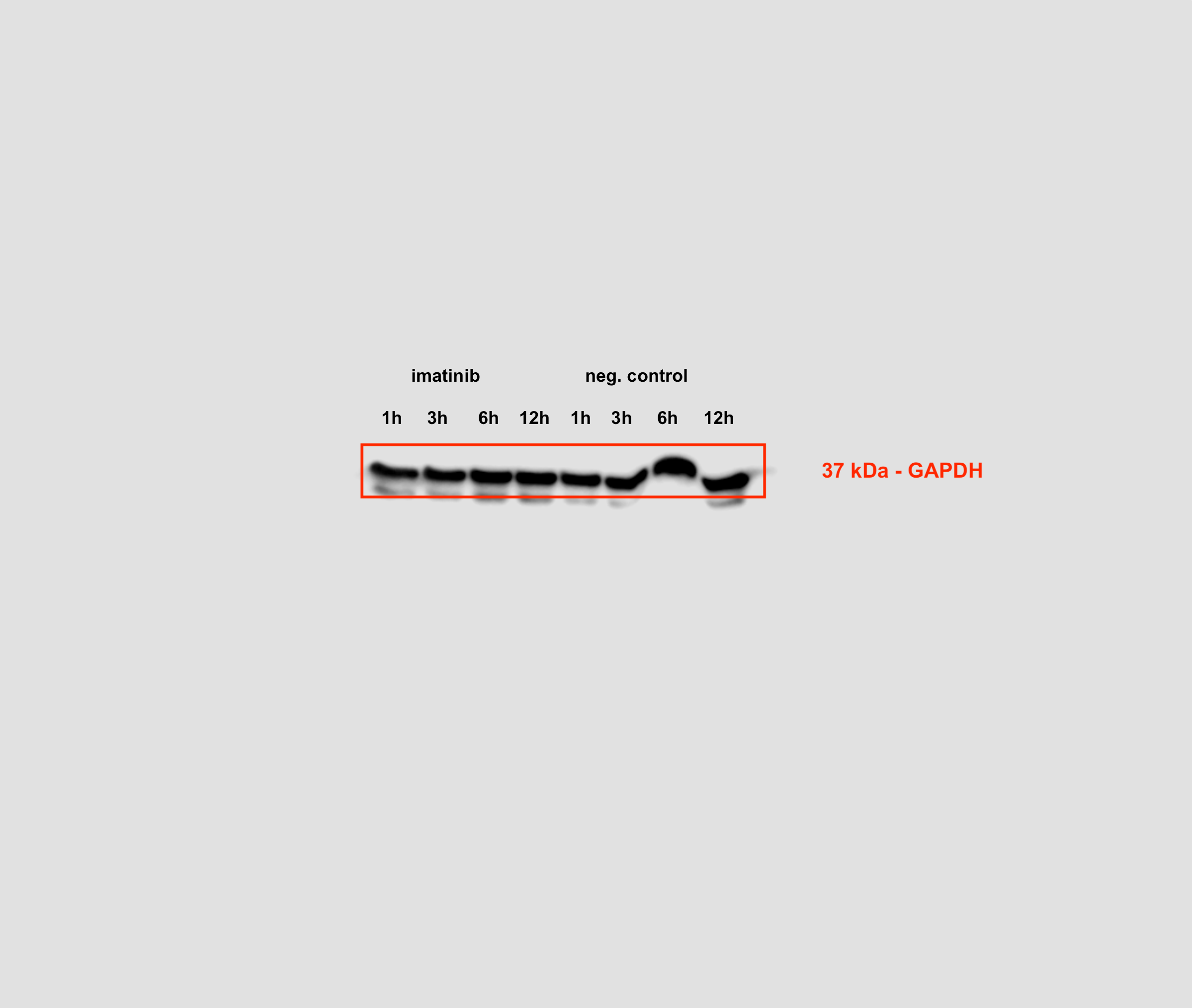

Supplement: Supplementary file 3 — Source data Fig. 5 [file 44320_2024_50_MOESM3_ESM.zip › Figure 5/5E/pCrkl/WB_19_231206_01_GAPDH_BCRABL1_1.tif]

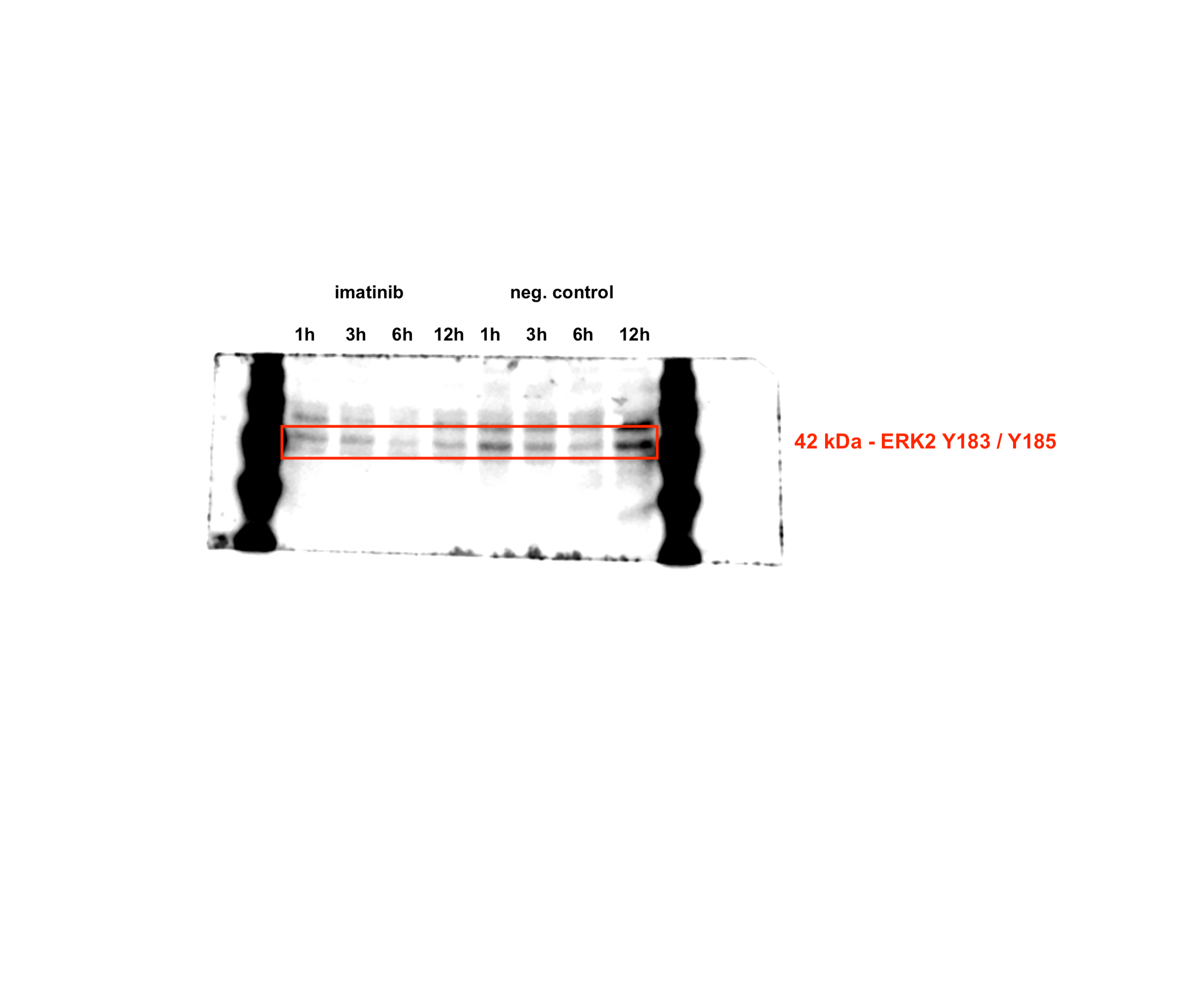

Supplement: Supplementary file 3 — Source data Fig. 5 [file 44320_2024_50_MOESM3_ESM.zip › Figure 5/5E/pERK/WB_13_231123_Imatinib_02_p-p44_p42_4.tif]

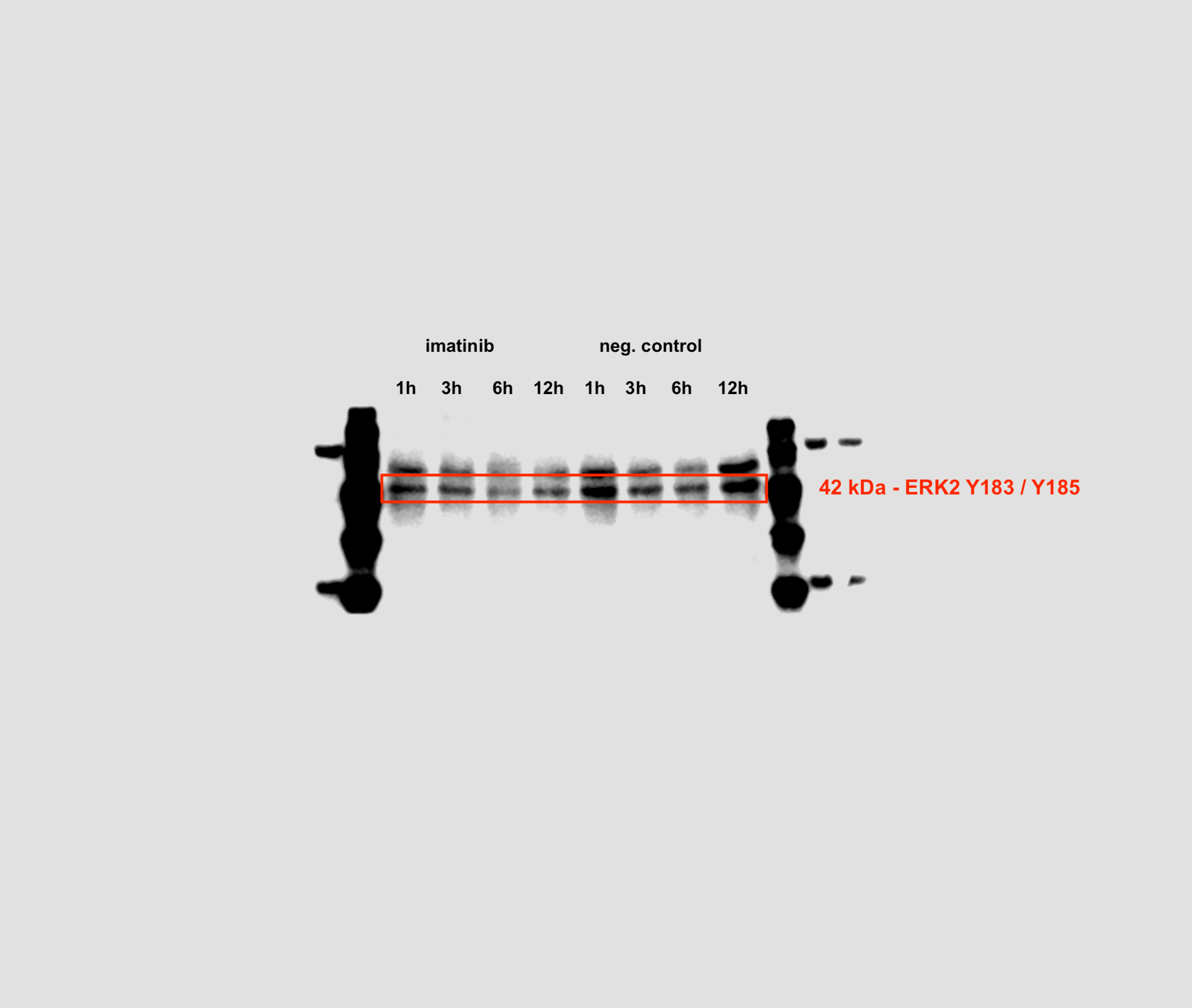

Supplement: Supplementary file 3 — Source data Fig. 5 [file 44320_2024_50_MOESM3_ESM.zip › Figure 5/5E/pERK/WB_15_231128_02_p-p44_p42_BaF3_BCRABL1_2.tif]

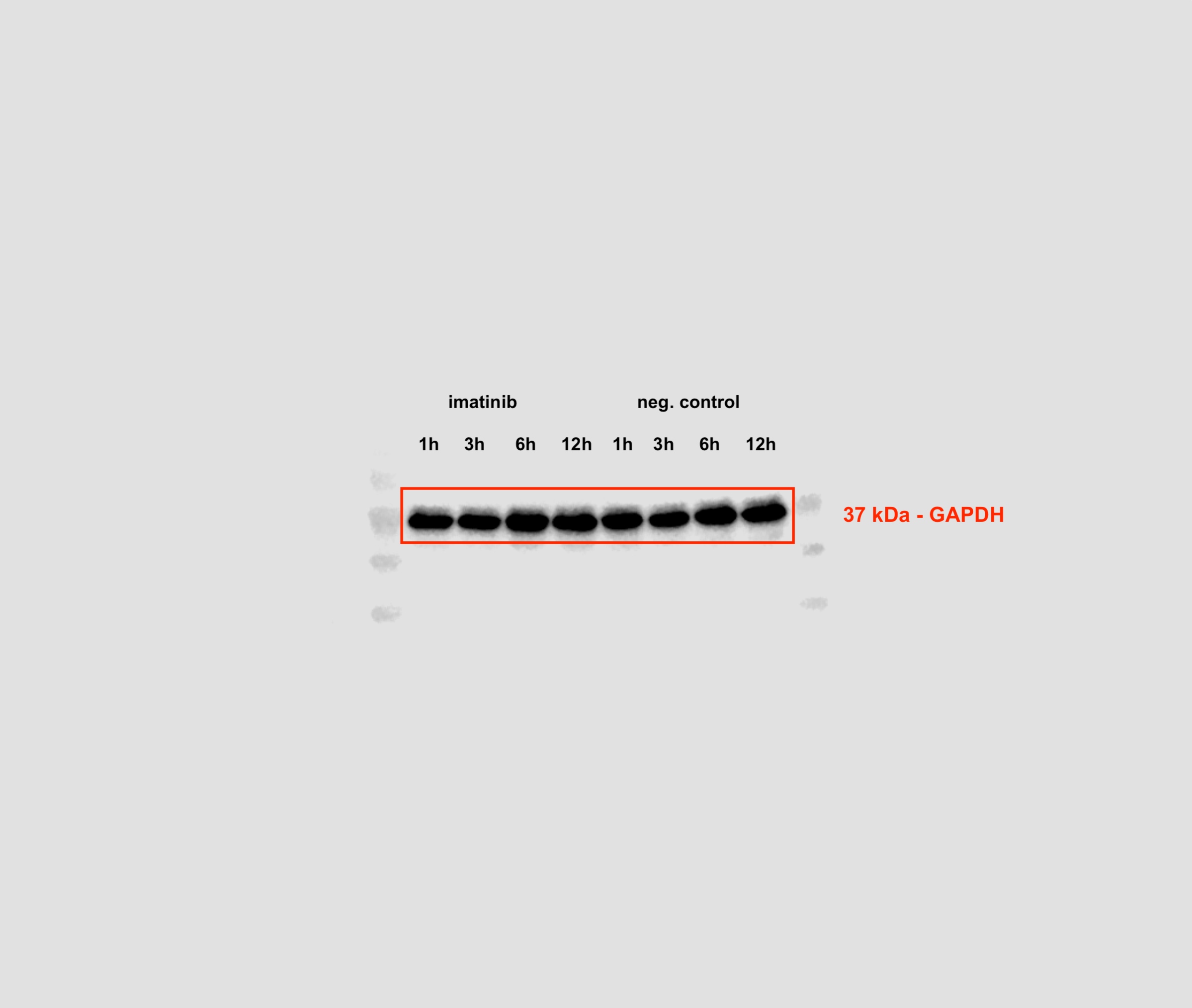

Supplement: Supplementary file 3 — Source data Fig. 5 [file 44320_2024_50_MOESM3_ESM.zip › Figure 5/5E/pERK/WB_15_231130_02_GAPDH_BaF3_BCRABL1_1.tif]

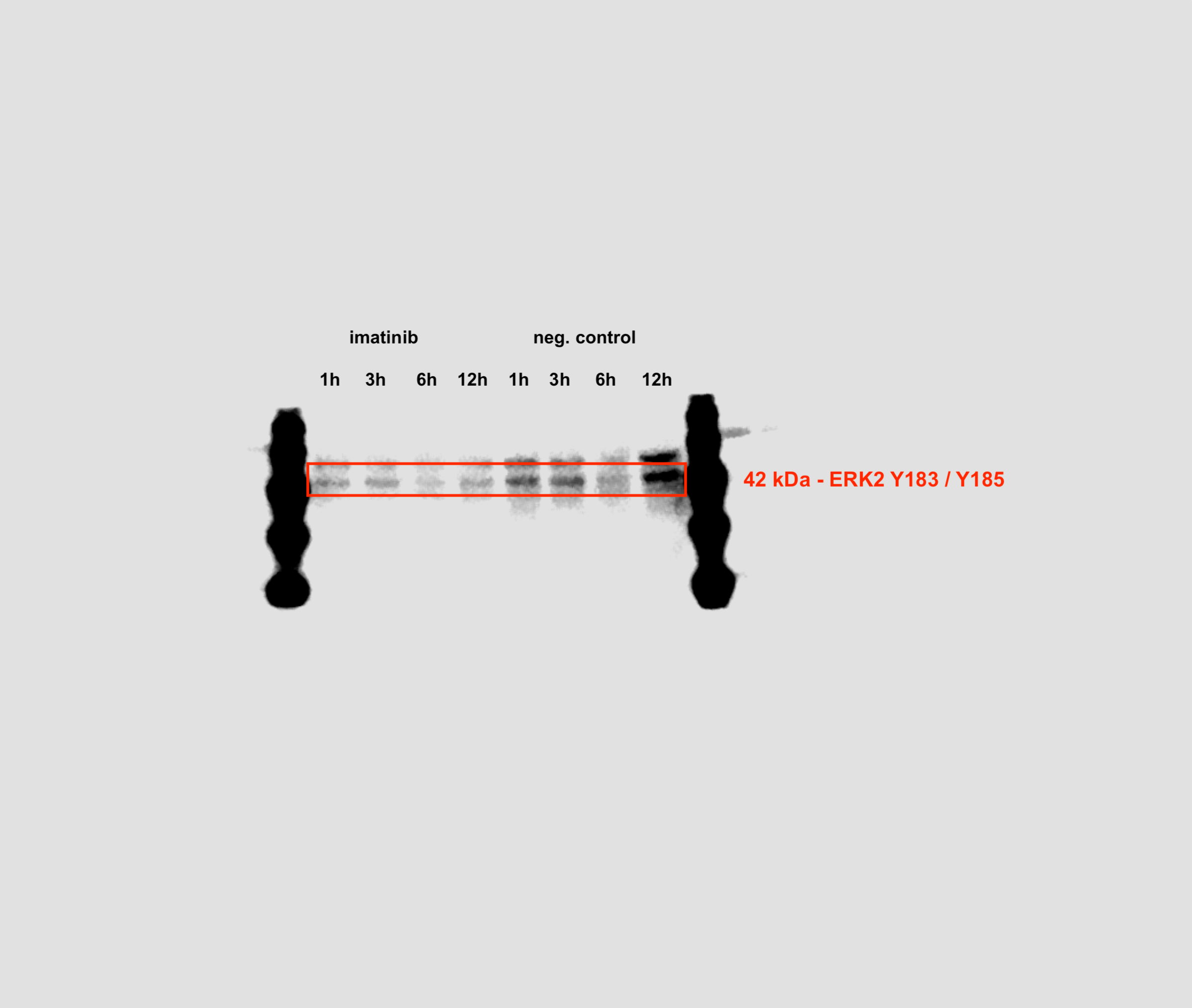

Supplement: Supplementary file 3 — Source data Fig. 5 [file 44320_2024_50_MOESM3_ESM.zip › Figure 5/5E/pERK/WB_17_231129_02_pERK_BaF3_BCRABL1_1.tif]

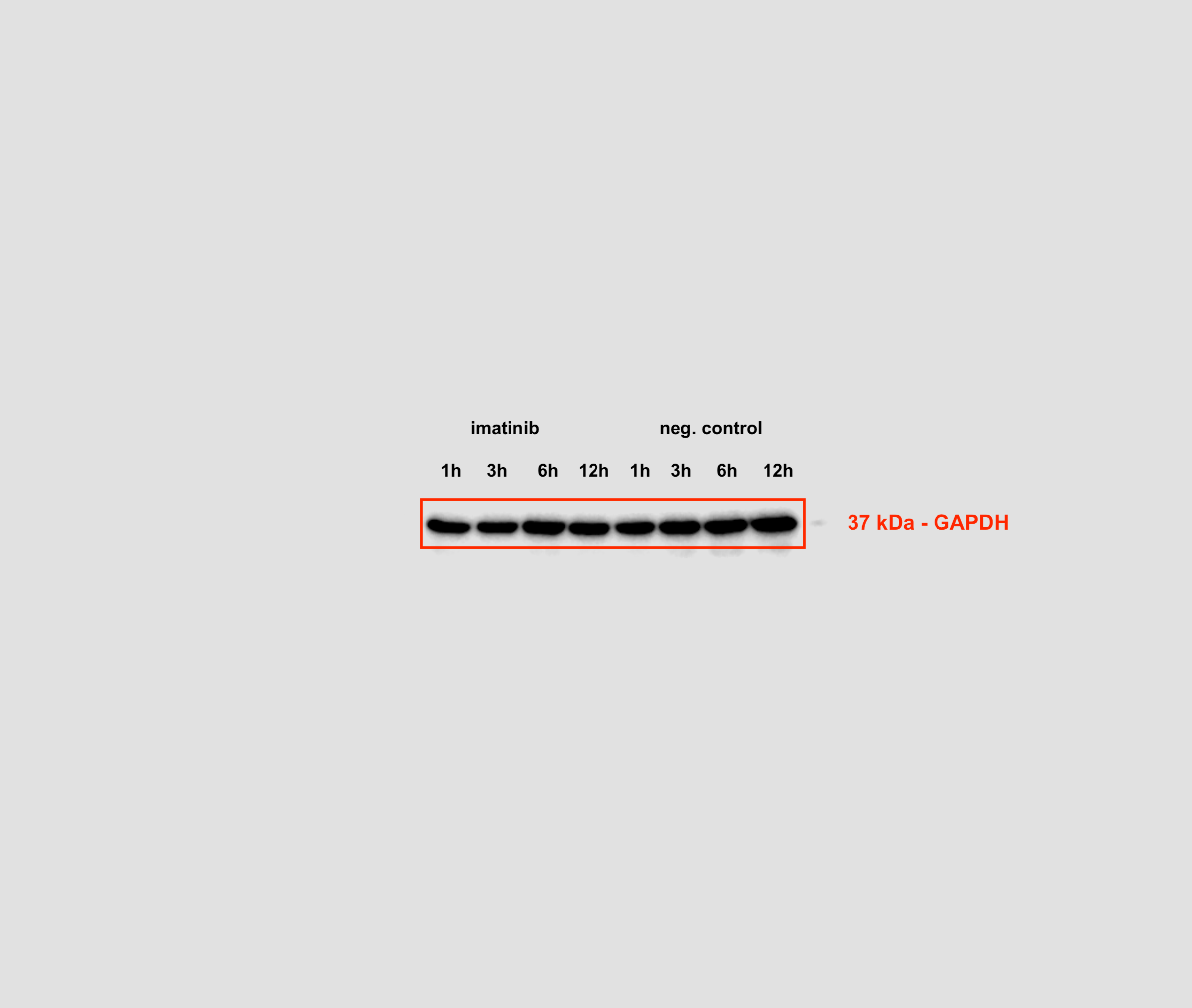

Supplement: Supplementary file 3 — Source data Fig. 5 [file 44320_2024_50_MOESM3_ESM.zip › Figure 5/5E/pERK/WB_17_231201_01_GAPDH_BaF3_BCRABL1_1.tif]

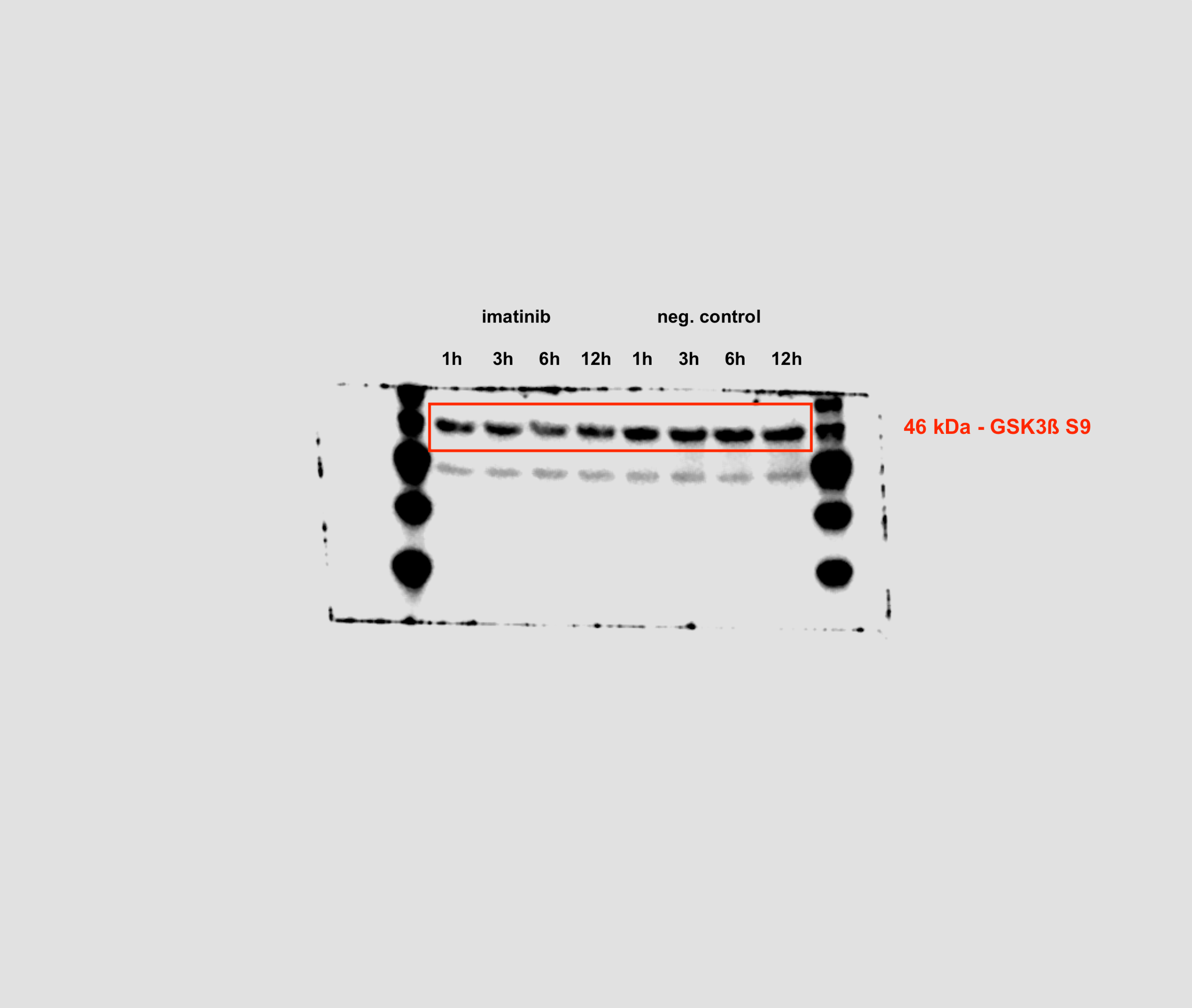

Supplement: Supplementary file 3 — Source data Fig. 5 [file 44320_2024_50_MOESM3_ESM.zip › Figure 5/5E/pGSK3b/WB_13_231123_Imatinib_01_pGSK3b_3.tif]

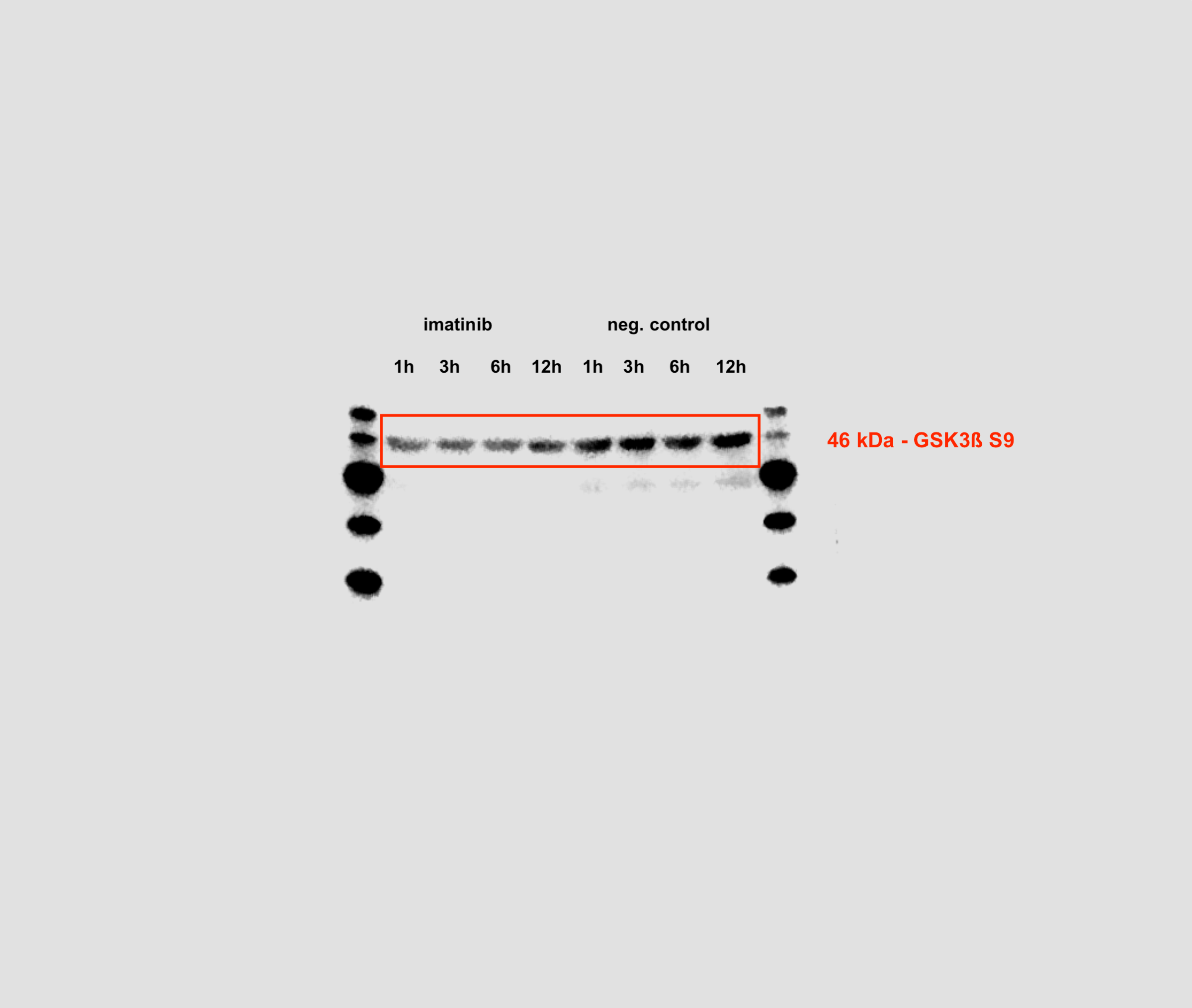

Supplement: Supplementary file 3 — Source data Fig. 5 [file 44320_2024_50_MOESM3_ESM.zip › Figure 5/5E/pGSK3b/WB_17_231129_01_pGSK3b_S9_BaF3_BCRABL1_2.tif]

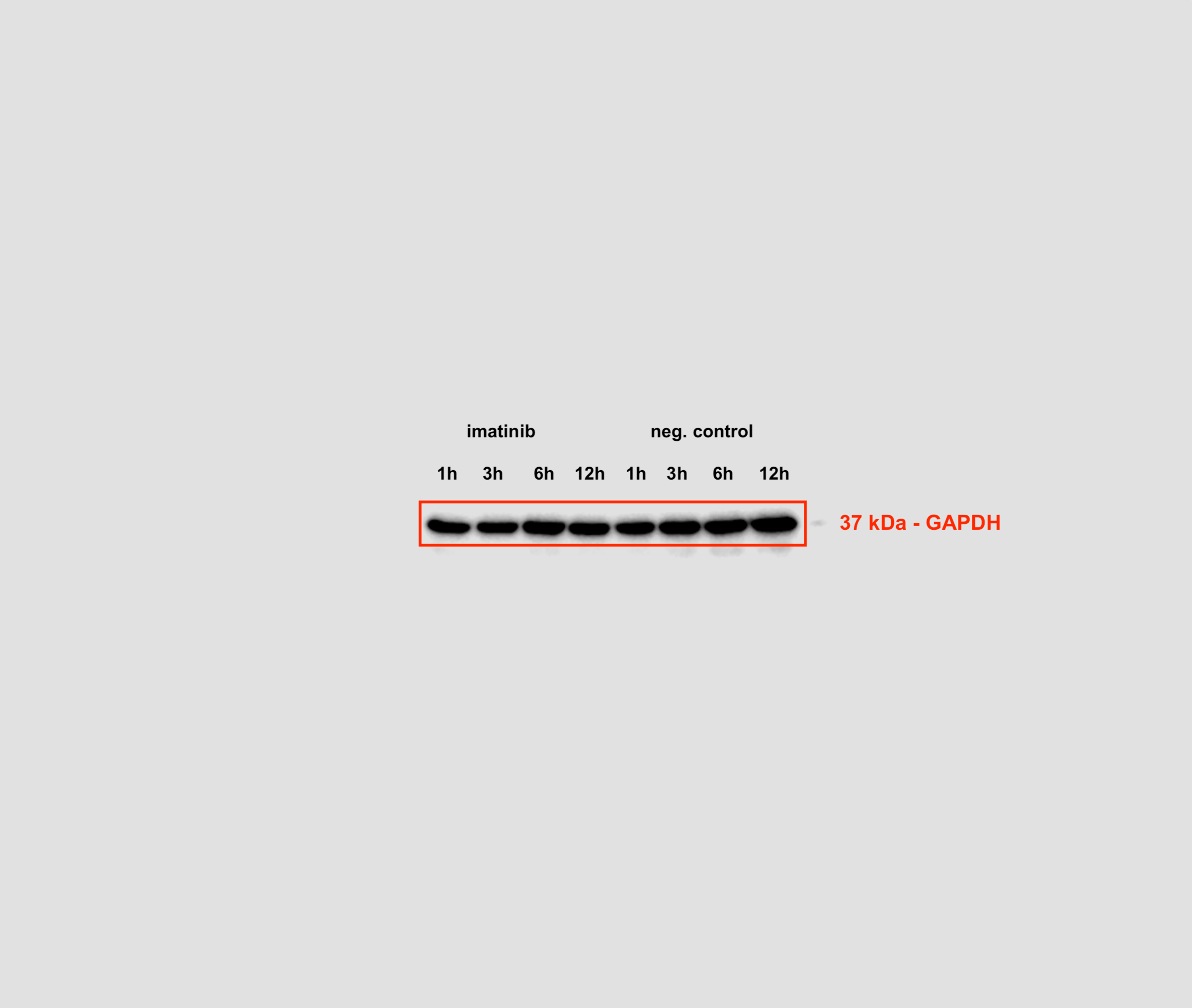

Supplement: Supplementary file 3 — Source data Fig. 5 [file 44320_2024_50_MOESM3_ESM.zip › Figure 5/5E/pGSK3b/WB_17_231201_01_GAPDH_BaF3_BCRABL1_1.tif]

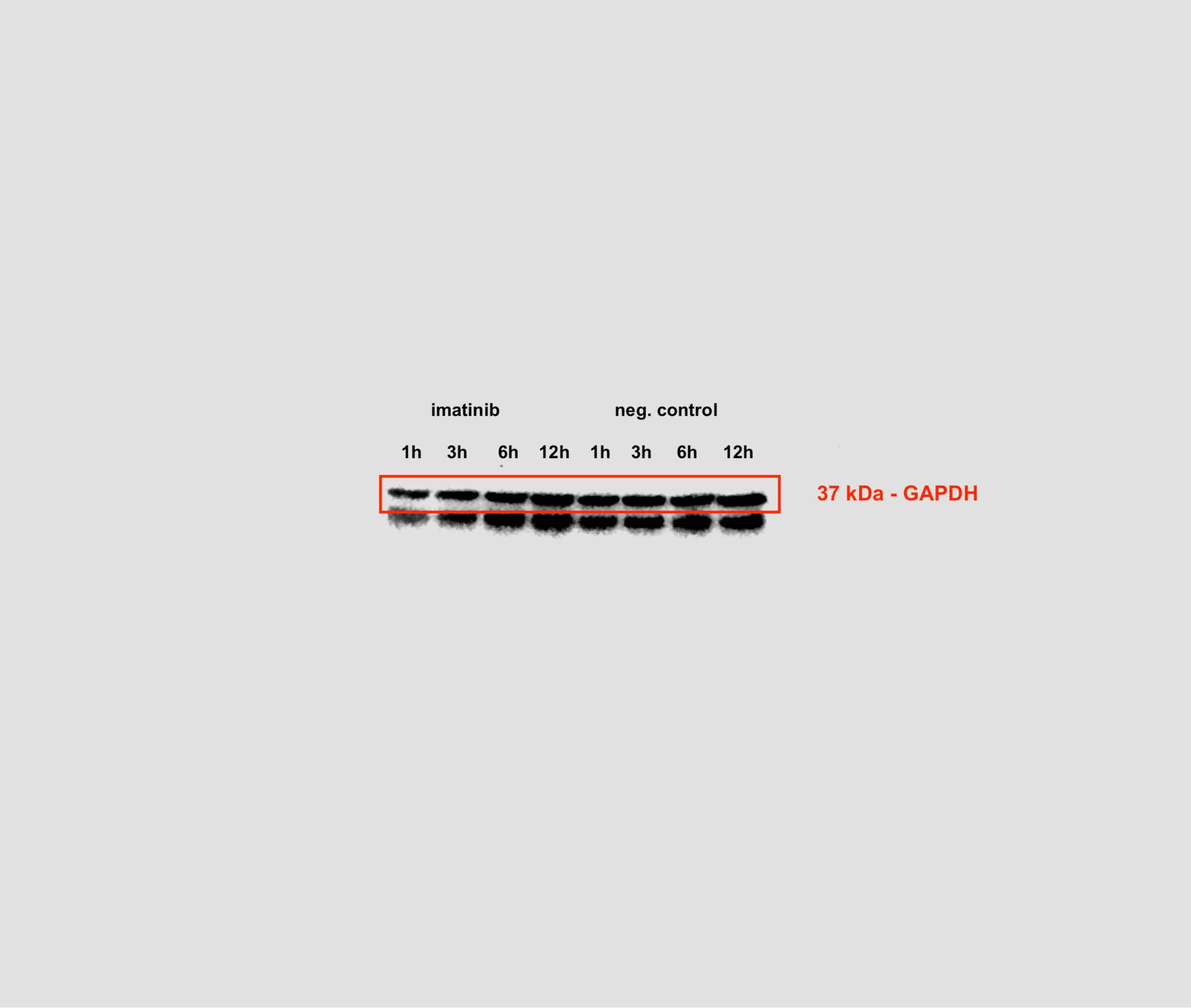

Supplement: Supplementary file 3 — Source data Fig. 5 [file 44320_2024_50_MOESM3_ESM.zip › Figure 5/5E/pGSK3b/WB_23_231215_GAPDH_BaF3-BCRABL1_Imatinib_1.tif]

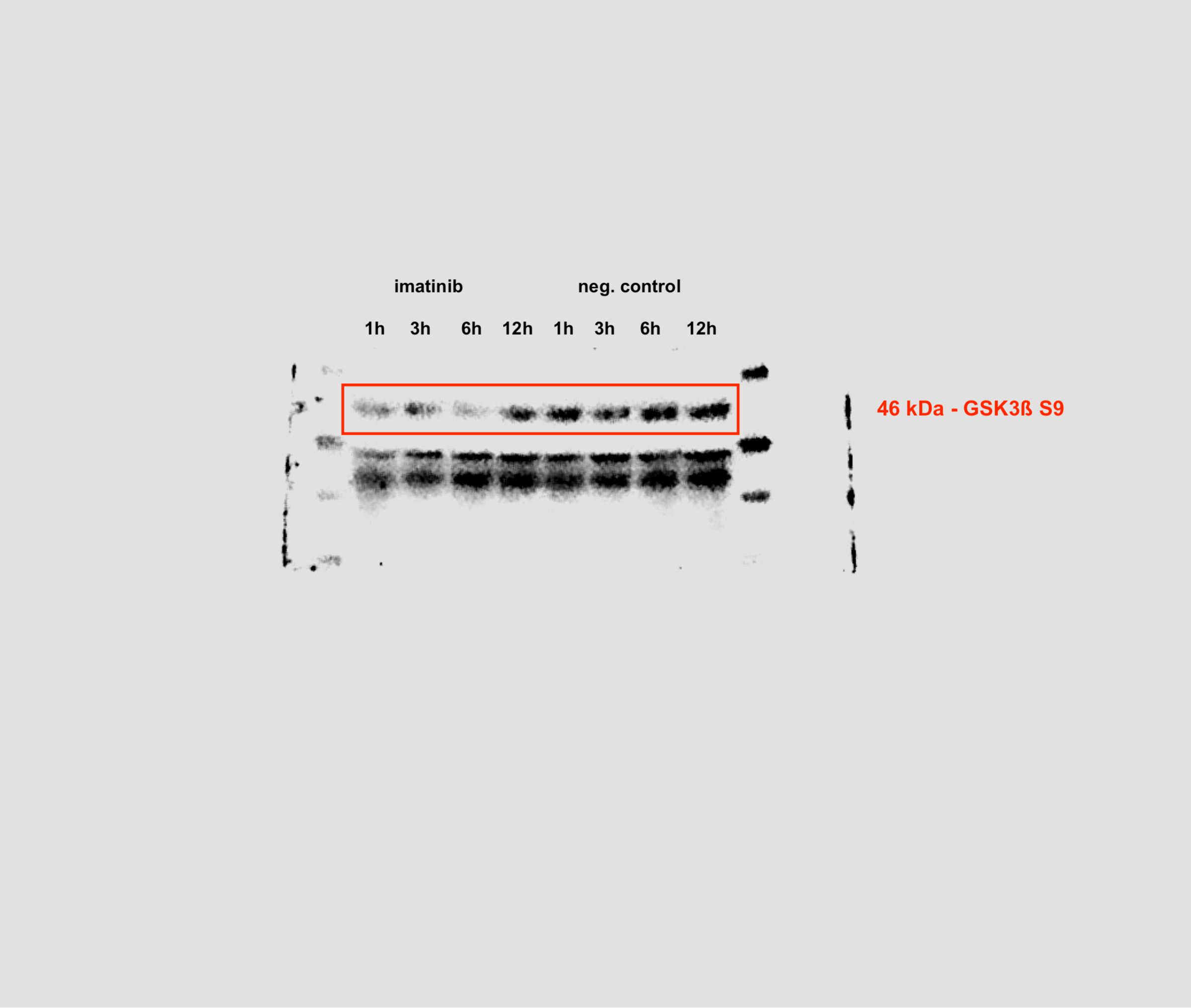

Supplement: Supplementary file 3 — Source data Fig. 5 [file 44320_2024_50_MOESM3_ESM.zip › Figure 5/5E/pGSK3b/WB_23_231215_pGSK3b_BaF3-BCRABL1_Imatinib_3.tif]

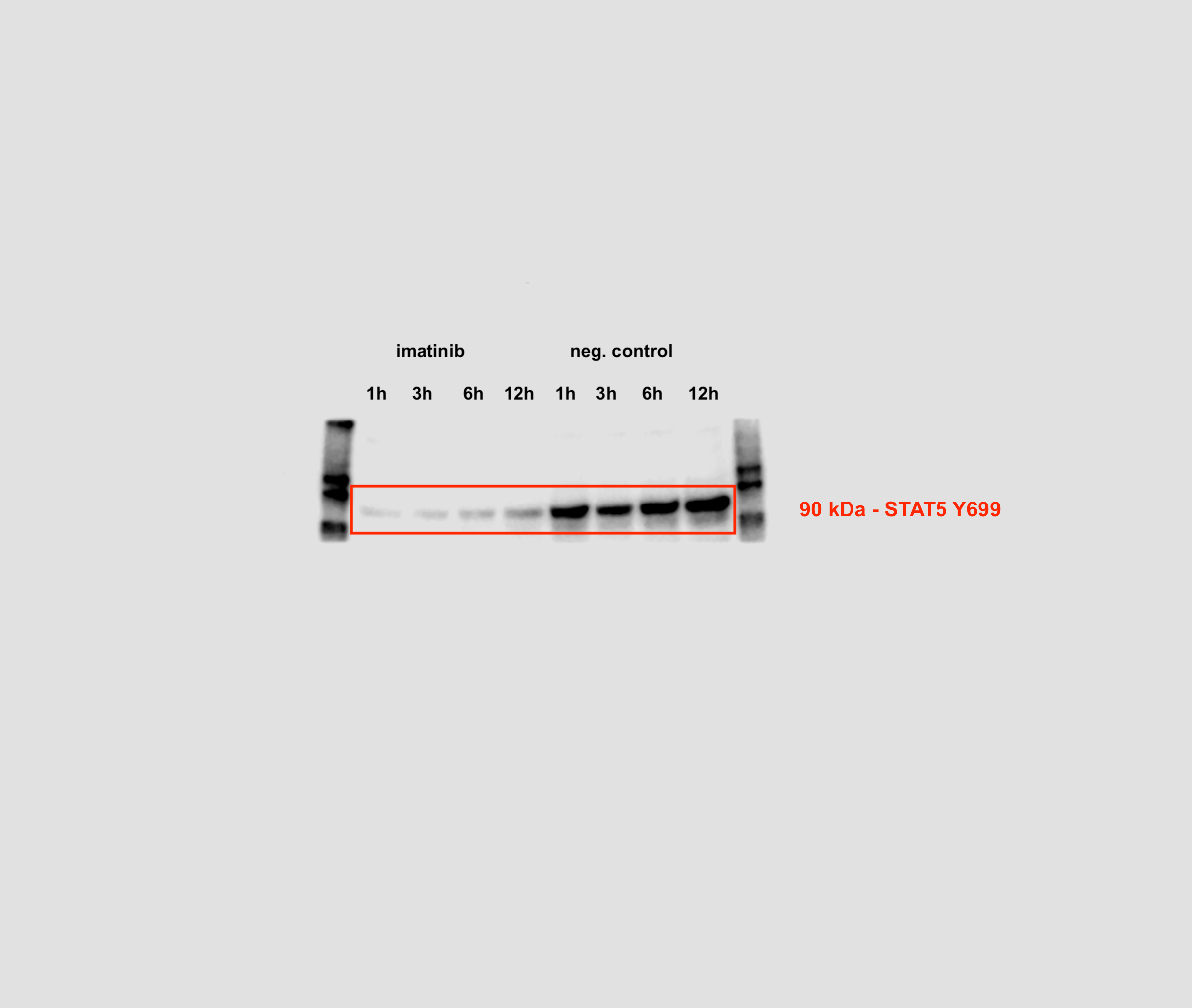

Supplement: Supplementary file 3 — Source data Fig. 5 [file 44320_2024_50_MOESM3_ESM.zip › Figure 5/5E/pStat5/WB_15_231129_02_pSTAT5-699_BaF3_BCRABL1_3.tif]

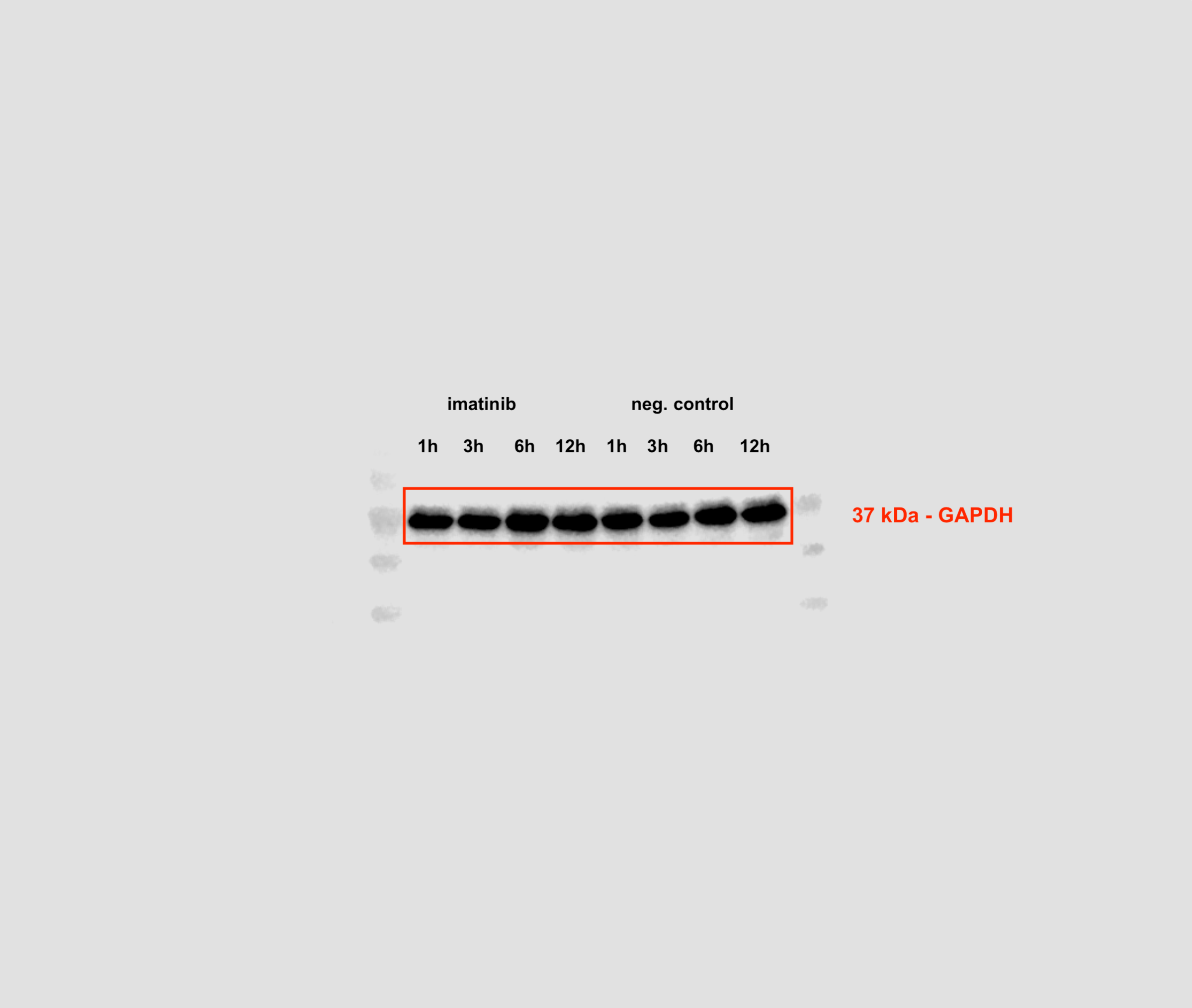

Supplement: Supplementary file 3 — Source data Fig. 5 [file 44320_2024_50_MOESM3_ESM.zip › Figure 5/5E/pStat5/WB_15_231130_02_GAPDH_BaF3_BCRABL1_1.tif]

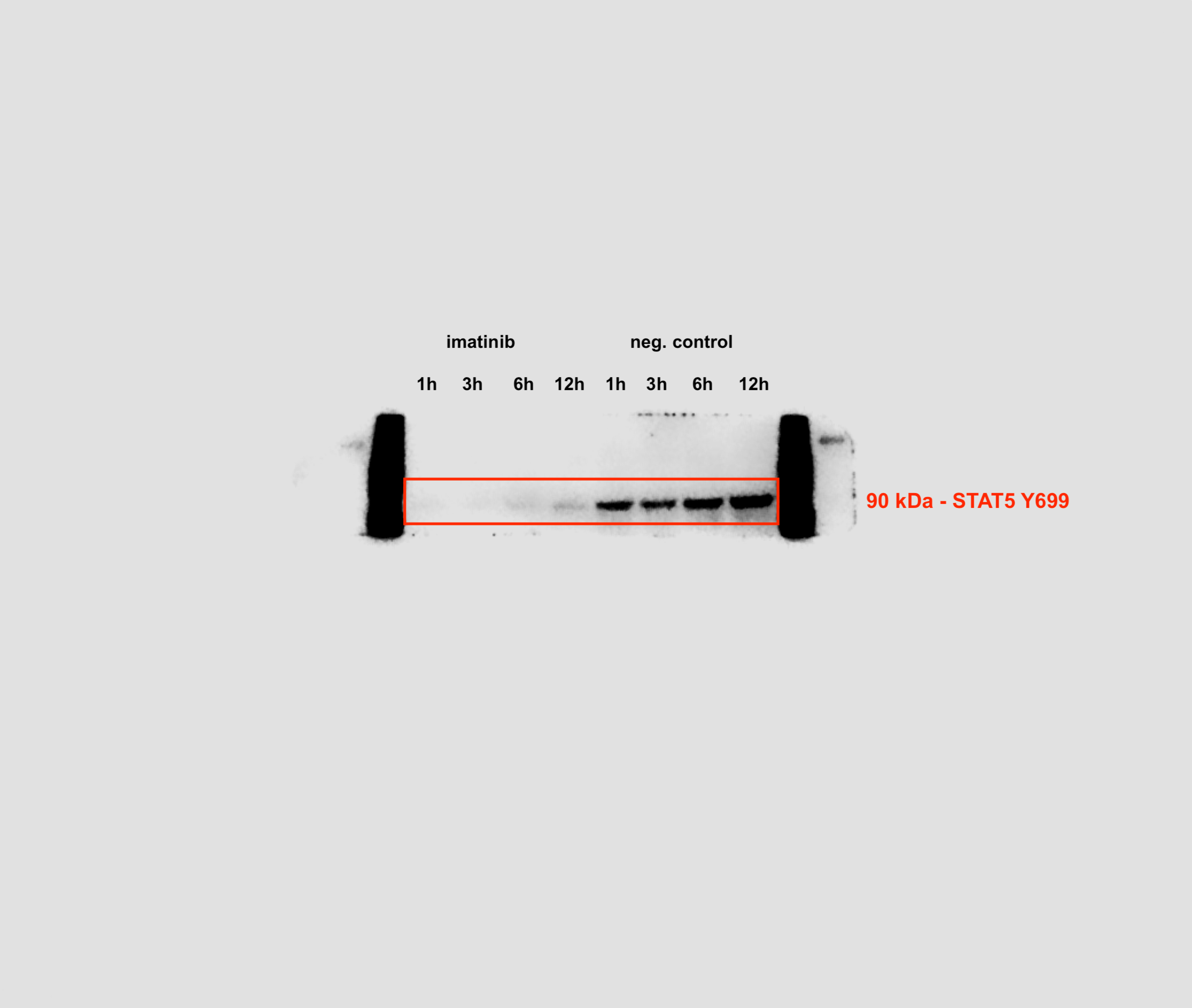

Supplement: Supplementary file 3 — Source data Fig. 5 [file 44320_2024_50_MOESM3_ESM.zip › Figure 5/5E/pStat5/WB_17_231129_01_pSTAT5_699_BaF3_BCRABL1_2.tif]

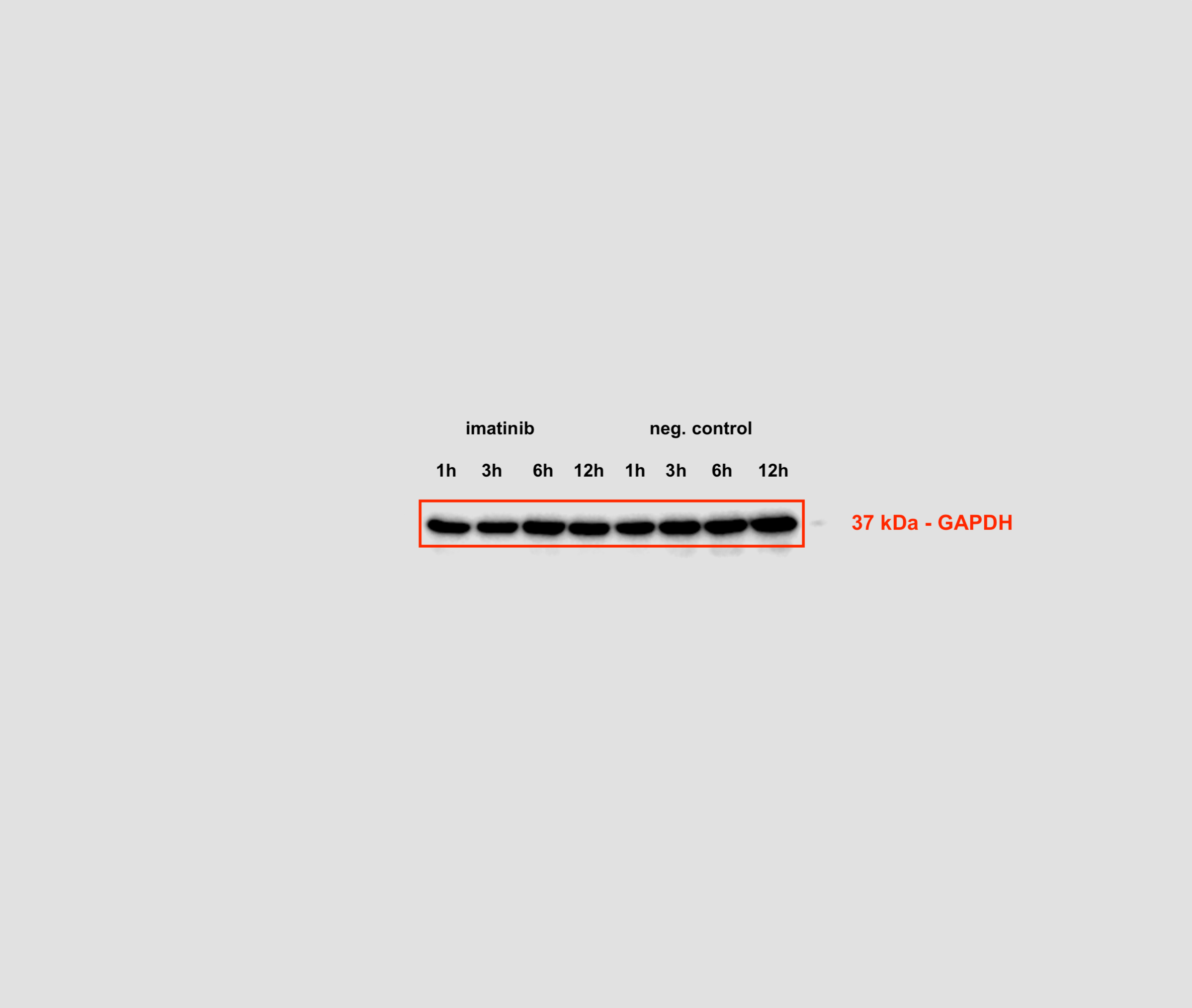

Supplement: Supplementary file 3 — Source data Fig. 5 [file 44320_2024_50_MOESM3_ESM.zip › Figure 5/5E/pStat5/WB_17_231201_01_GAPDH_BaF3_BCRABL1_1.tif]

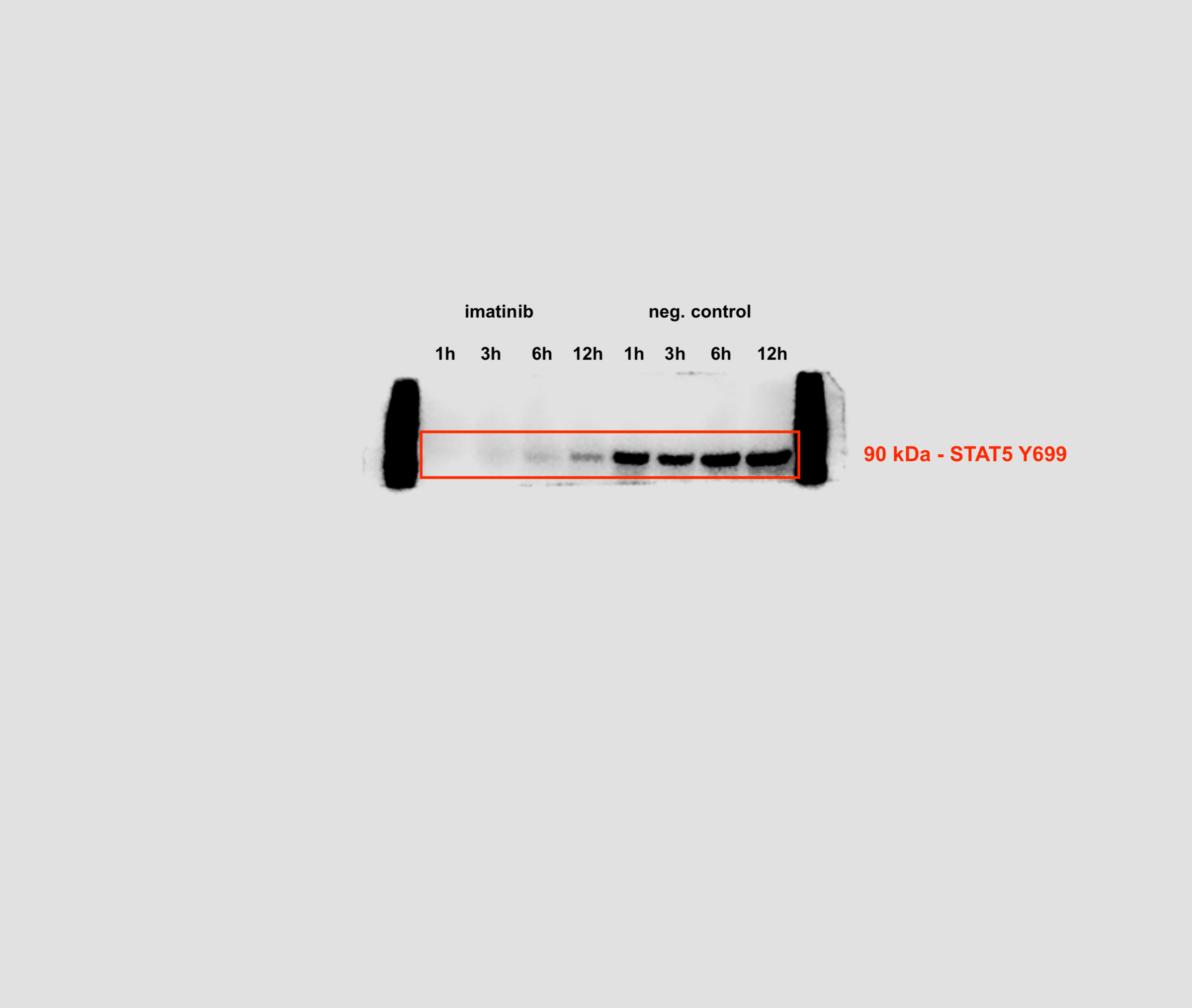

Supplement: Supplementary file 3 — Source data Fig. 5 [file 44320_2024_50_MOESM3_ESM.zip › Figure 5/5E/pStat5/WB_20_231205_01_pSTAT5_BaF3_BCRABL1_4.tif]

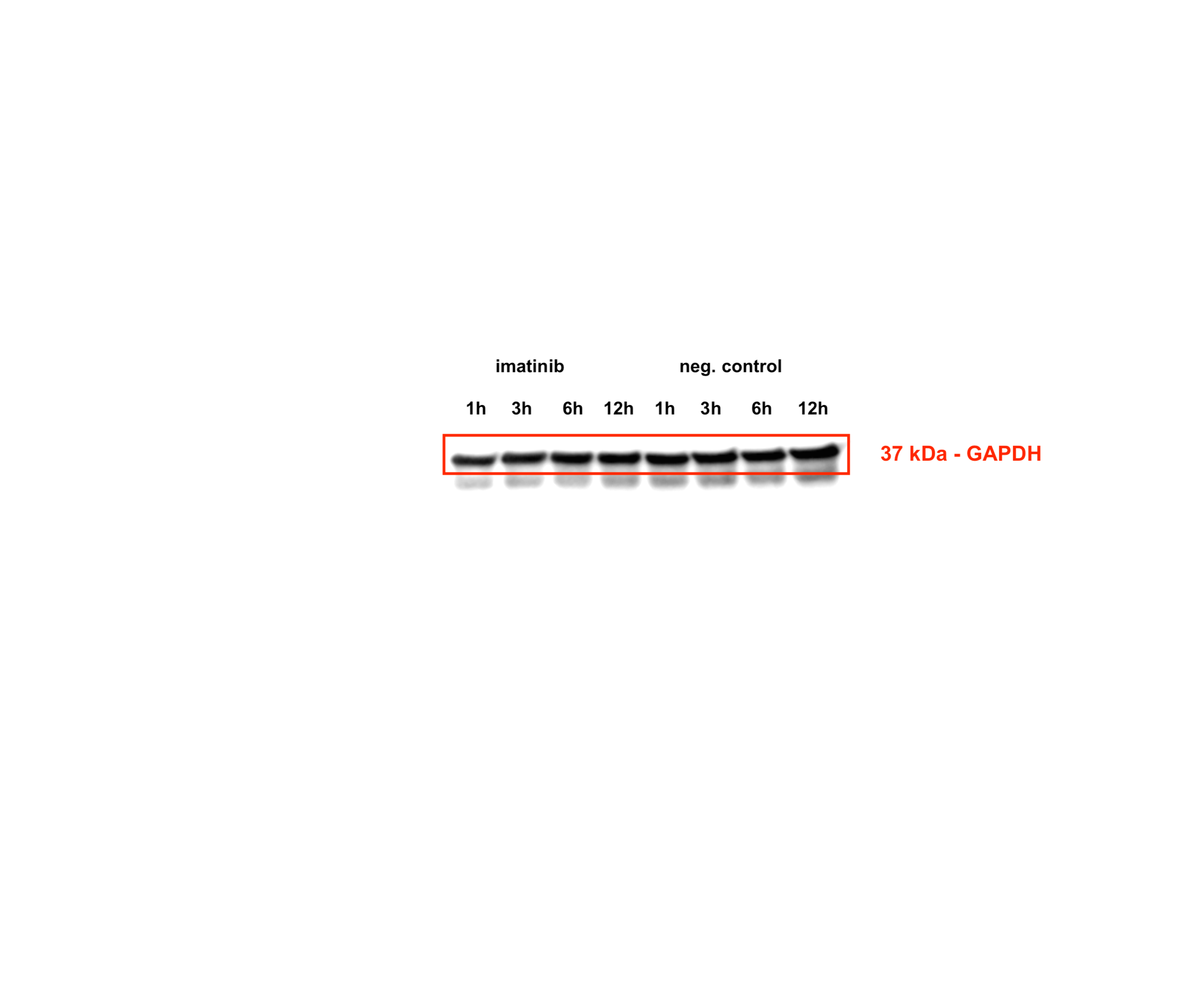

Supplement: Supplementary file 3 — Source data Fig. 5 [file 44320_2024_50_MOESM3_ESM.zip › Figure 5/5E/pStat5/WB_20_231207_GAPDH_BaF3_BCRABL1_3.tif]

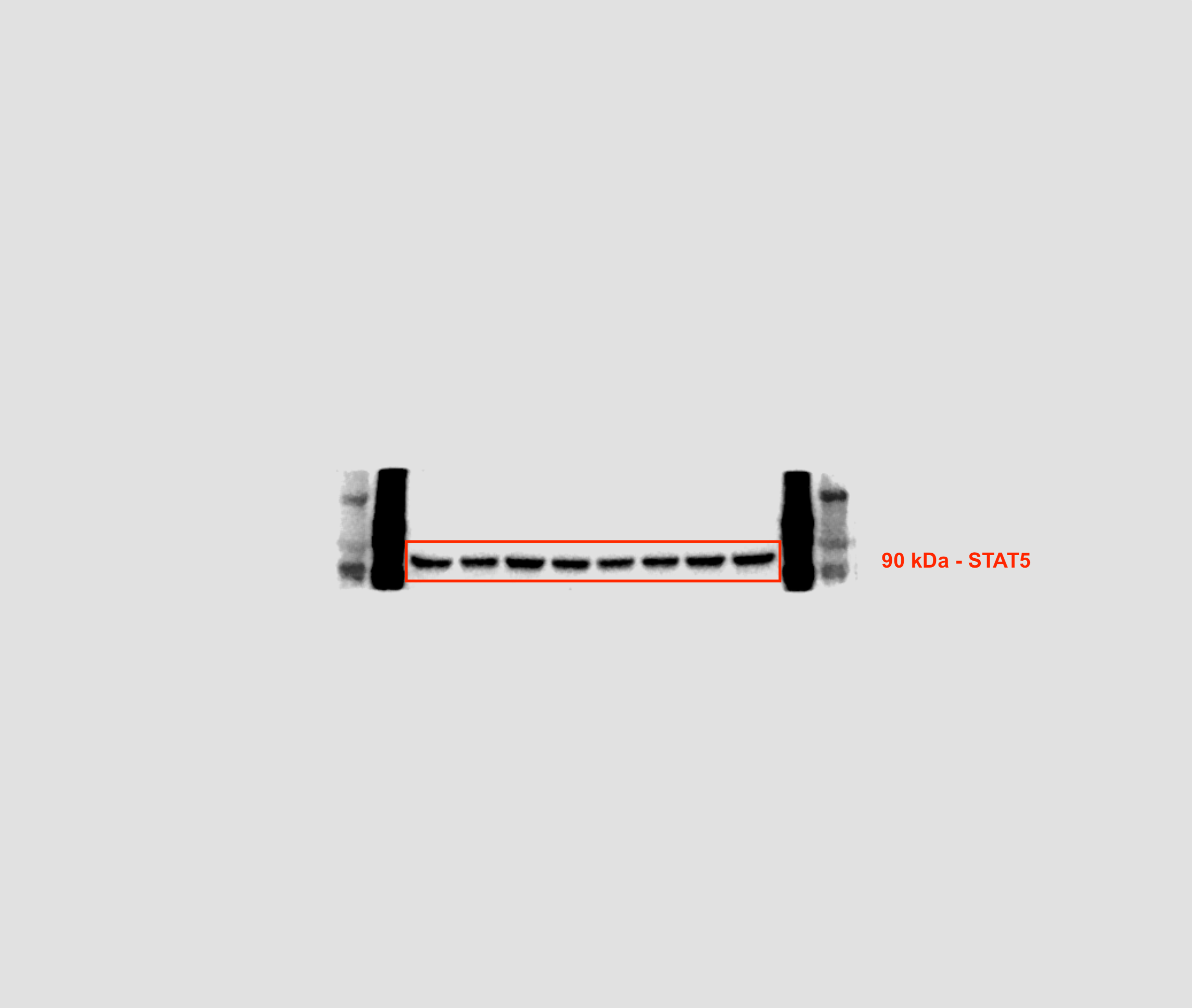

Supplement: Supplementary file 3 — Source data Fig. 5 [file 44320_2024_50_MOESM3_ESM.zip › Figure 5/5E/Stat5/WB_17_231130_01_STAT5_BaF3_BCRABL1_4.tif]

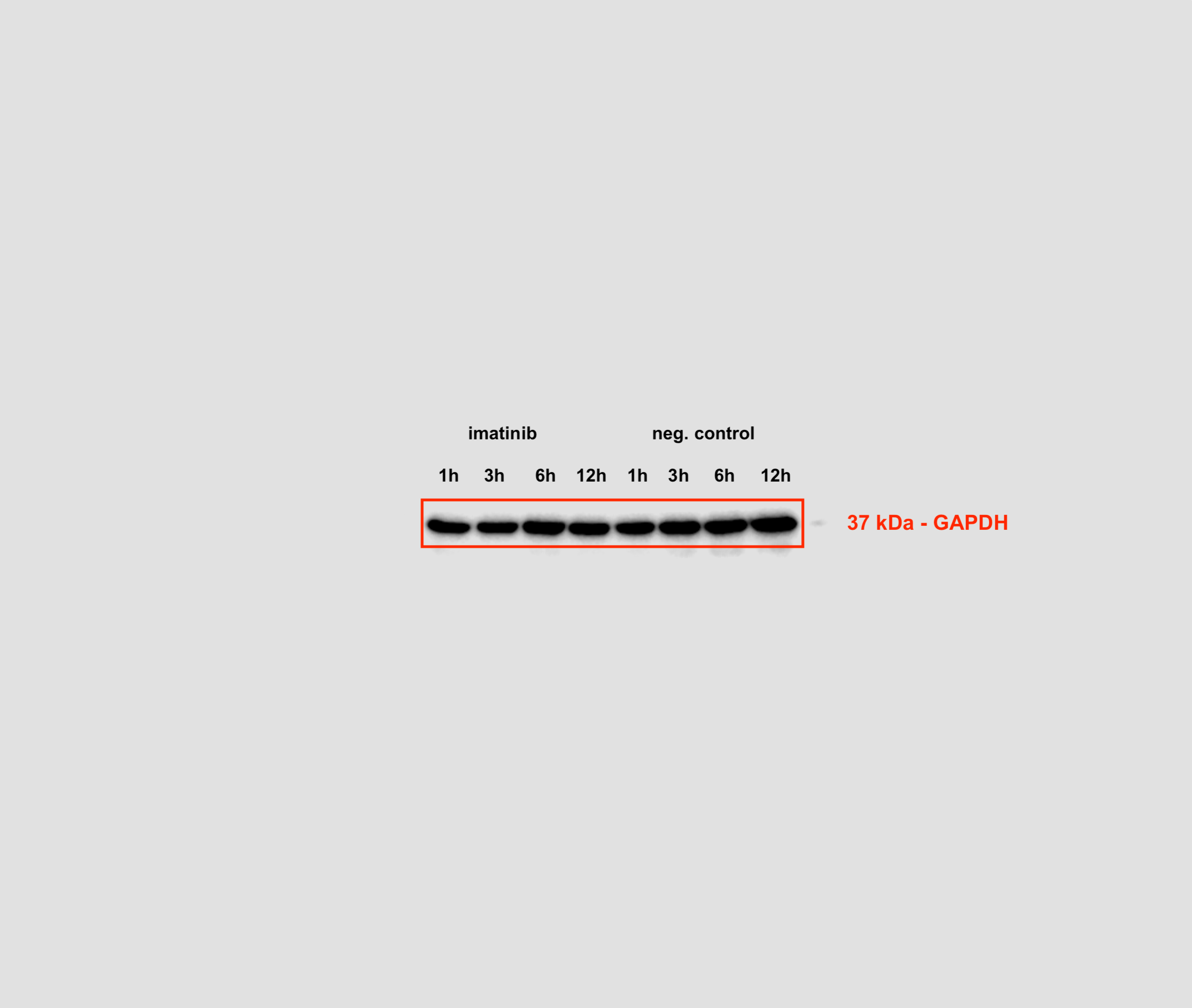

Supplement: Supplementary file 3 — Source data Fig. 5 [file 44320_2024_50_MOESM3_ESM.zip › Figure 5/5E/Stat5/WB_17_231201_01_GAPDH_BaF3_BCRABL1_1.tif]

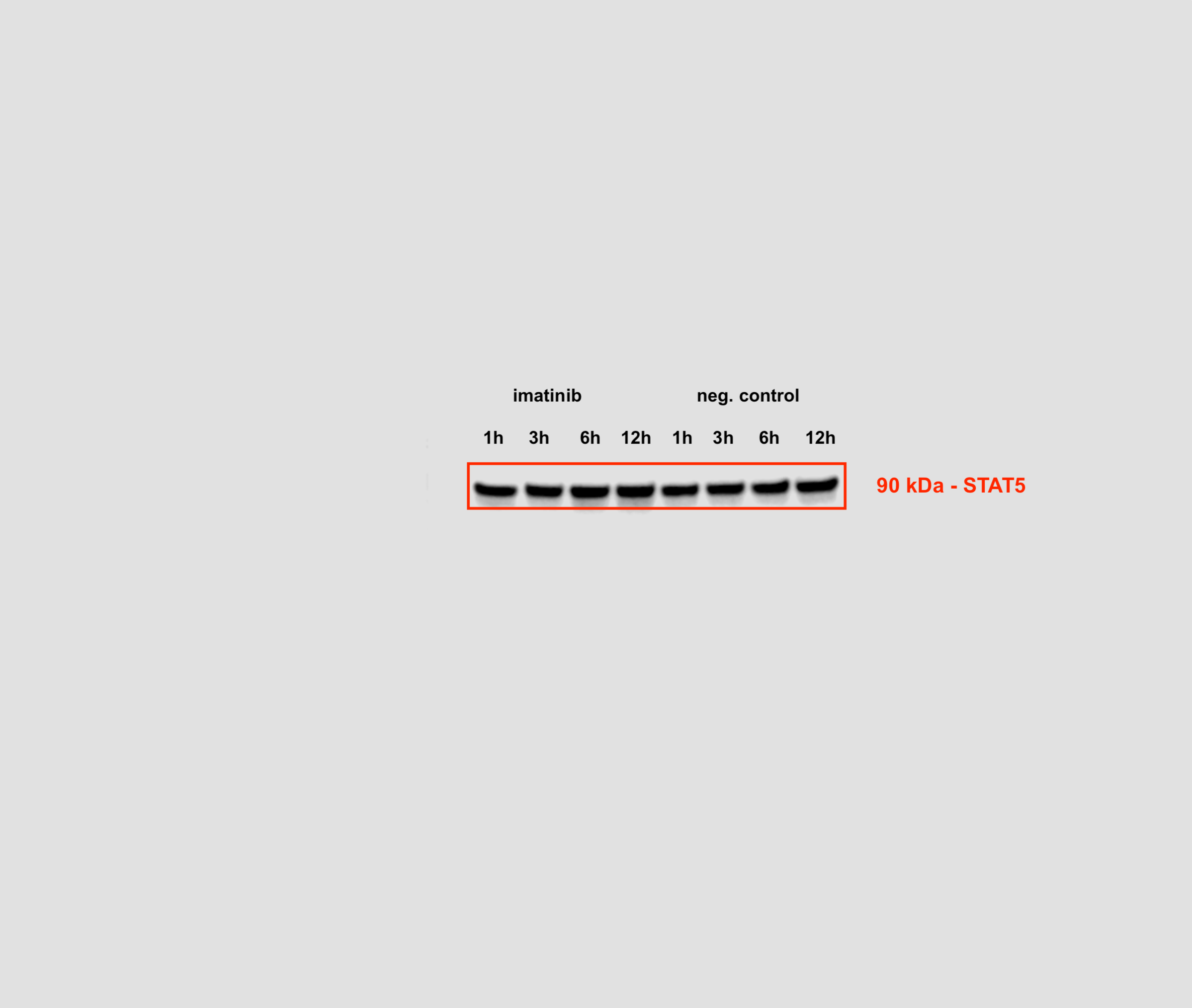

Supplement: Supplementary file 3 — Source data Fig. 5 [file 44320_2024_50_MOESM3_ESM.zip › Figure 5/5E/Stat5/WB_19_231205_STAT5_BaF3_BCRABL1_1.tif]

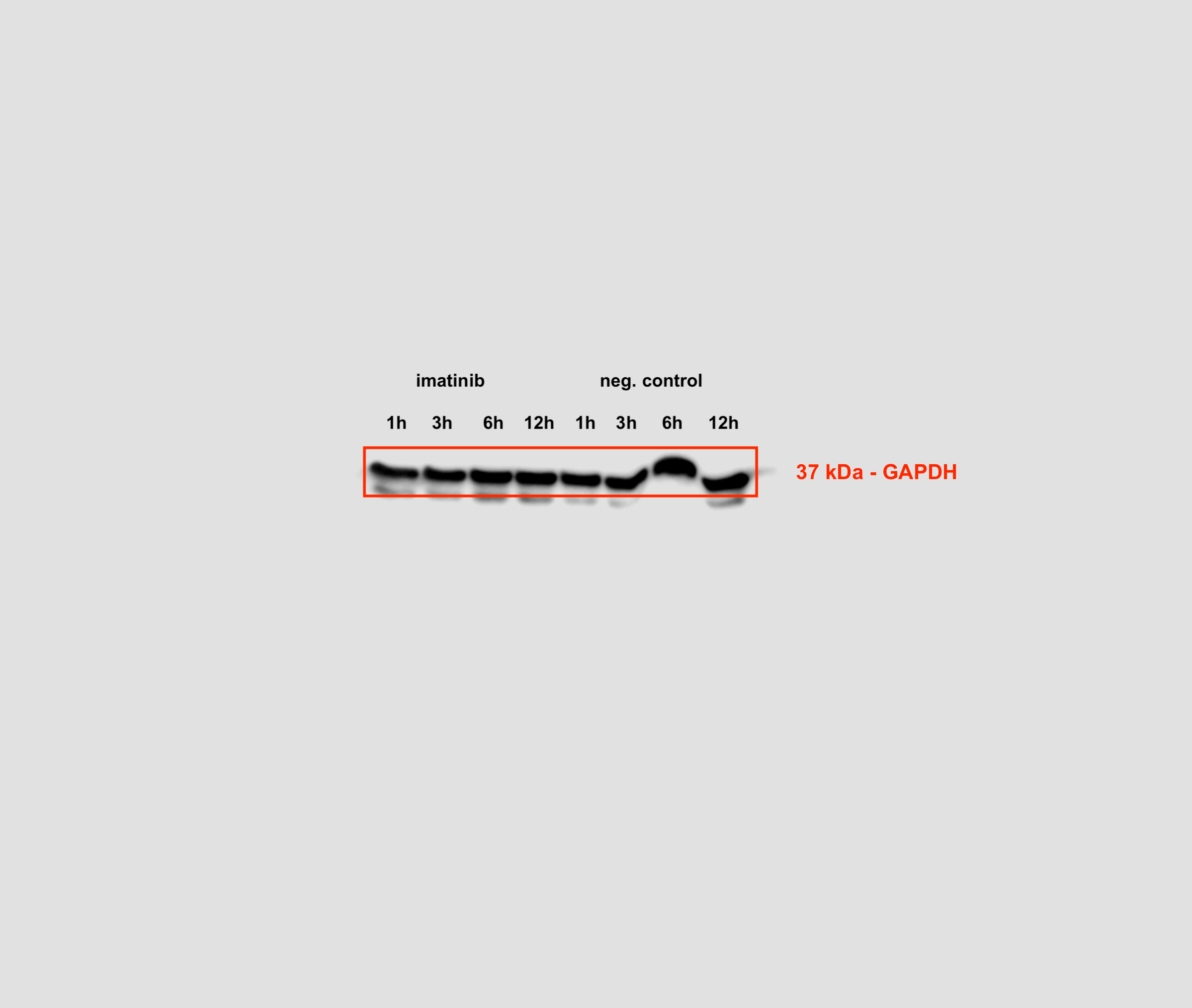

Supplement: Supplementary file 3 — Source data Fig. 5 [file 44320_2024_50_MOESM3_ESM.zip › Figure 5/5E/Stat5/WB_19_231206_01_GAPDH_BCRABL1_1.tif]

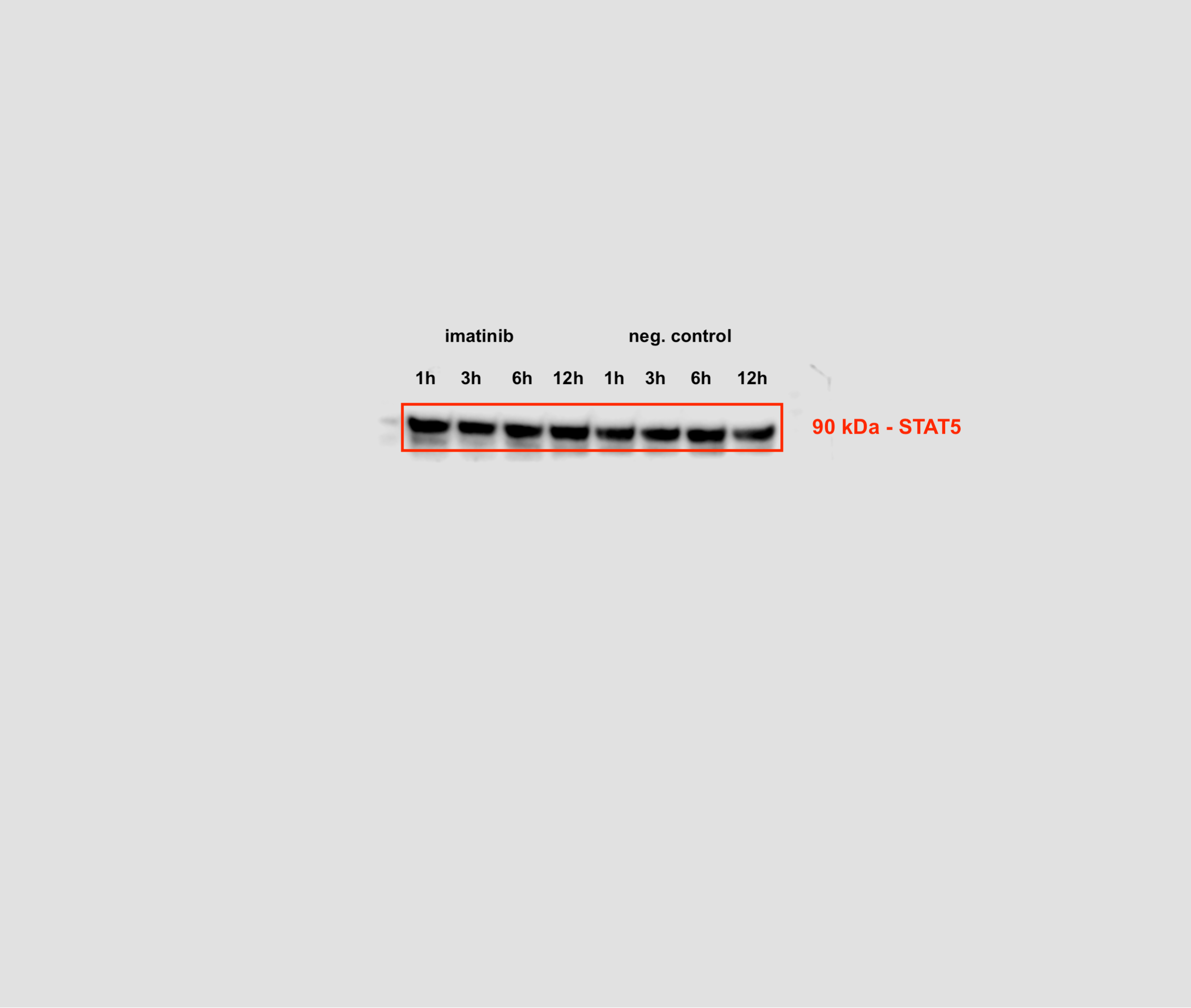

Supplement: Supplementary file 3 — Source data Fig. 5 [file 44320_2024_50_MOESM3_ESM.zip › Figure 5/5E/Stat5/WB_21_231213_STAT5_BaF3-BCRABL1_Imatinib_1.tif]

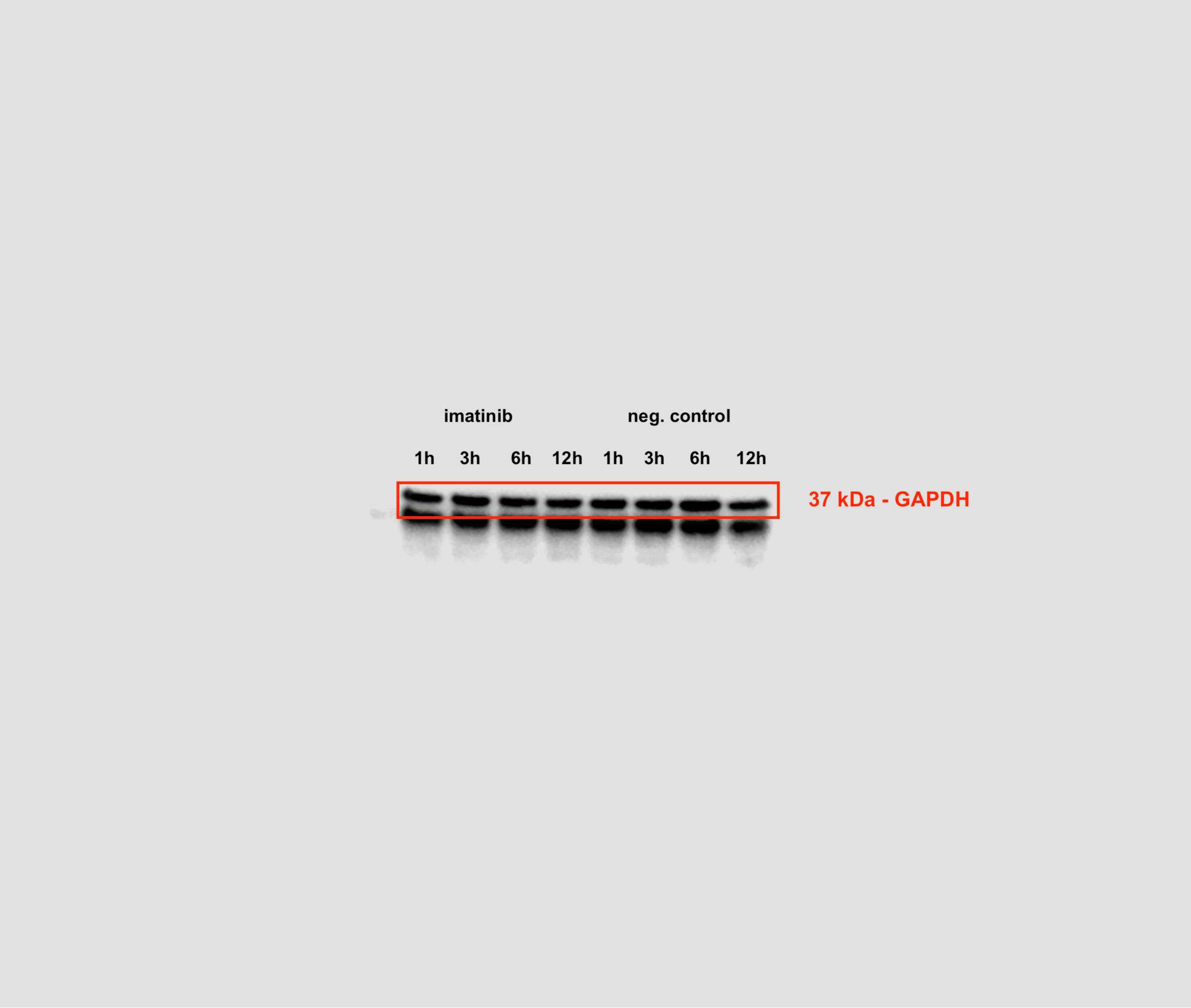

Supplement: Supplementary file 3 — Source data Fig. 5 [file 44320_2024_50_MOESM3_ESM.zip › Figure 5/5E/Stat5/WB_21_231214_GAPDH_BaF3-BCRABL1_Imatinib_1.tif]
