# Supplementary material for: µPhos: a scalable and sensitive platform for high-dimensional phosphoproteomics
Source: Mol Syst Biol. 2024 Jun 21;20(8):8. doi: 10.1038/s44320-024-00050-9 (PMC11297287; doi:10.1038/s44320-024-00050-9)
Supplement: Supplementary file 4 — Expanded View Figures [file 44320_2024_50_MOESM4_ESM.pdf]

Expanded View Figures

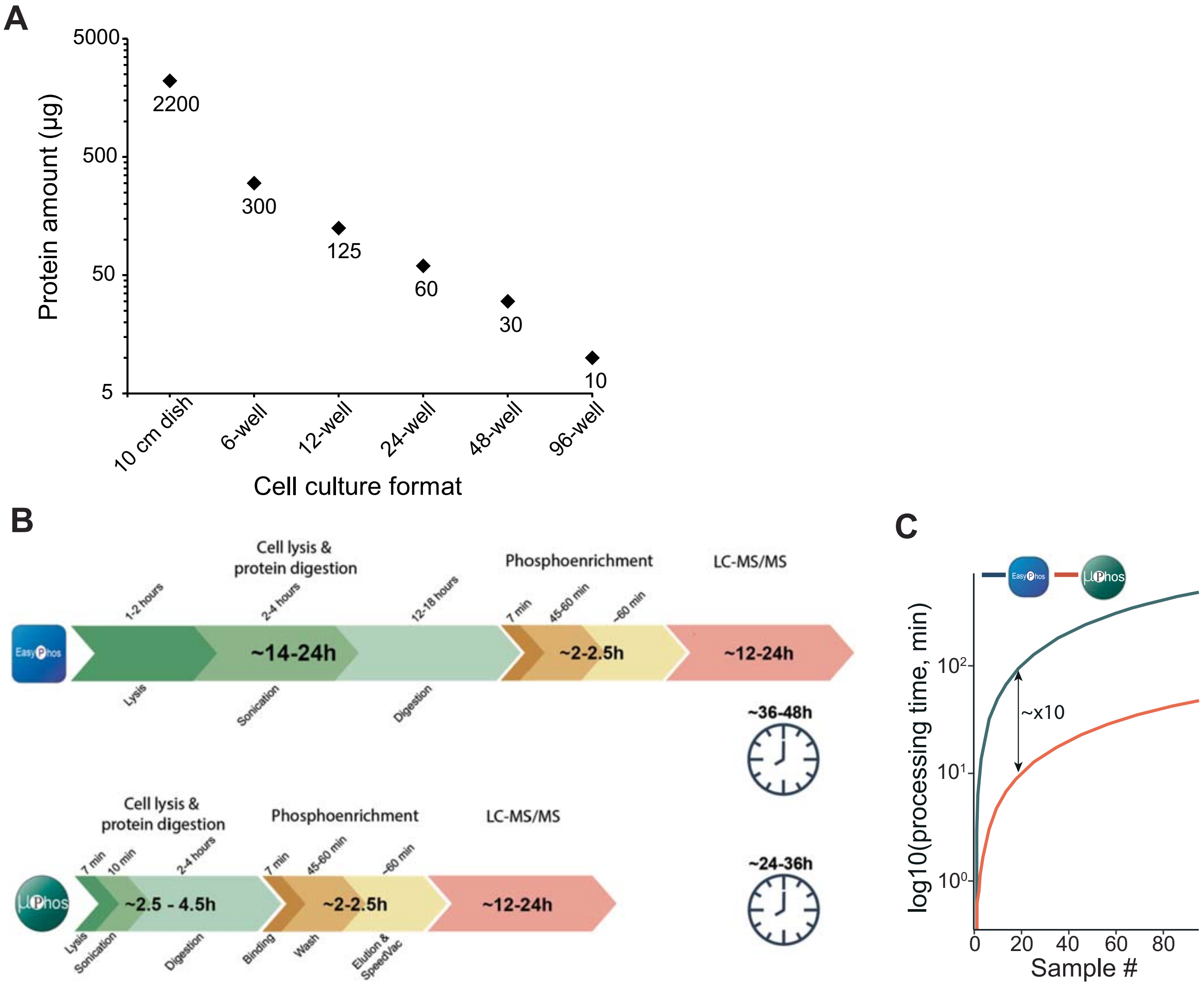

Figure EV1. Input amount and time considerations for large-scale phosphoproteomics.

(**A**) Protein input amounts (log scale) for different cell culture formats. Estimates are based on the number of HeLa cells at confluency and assuming on average 250 pg protein per cell. Data source: https://www.thermofisher.com/de/de/home/references/gibco-cell-culture-basics/cell-culture-protocols/cell-culture-useful-numbers.html (last accessed March 13, 2024). (**B**) Time scale of sample handling with the µPhos protocol compared to EasyPhos. (**C**) Estimation of the cumulative time-per-sample for the pre-digestion processing steps calculated from the average of 96 samples.

 © The Author(s)

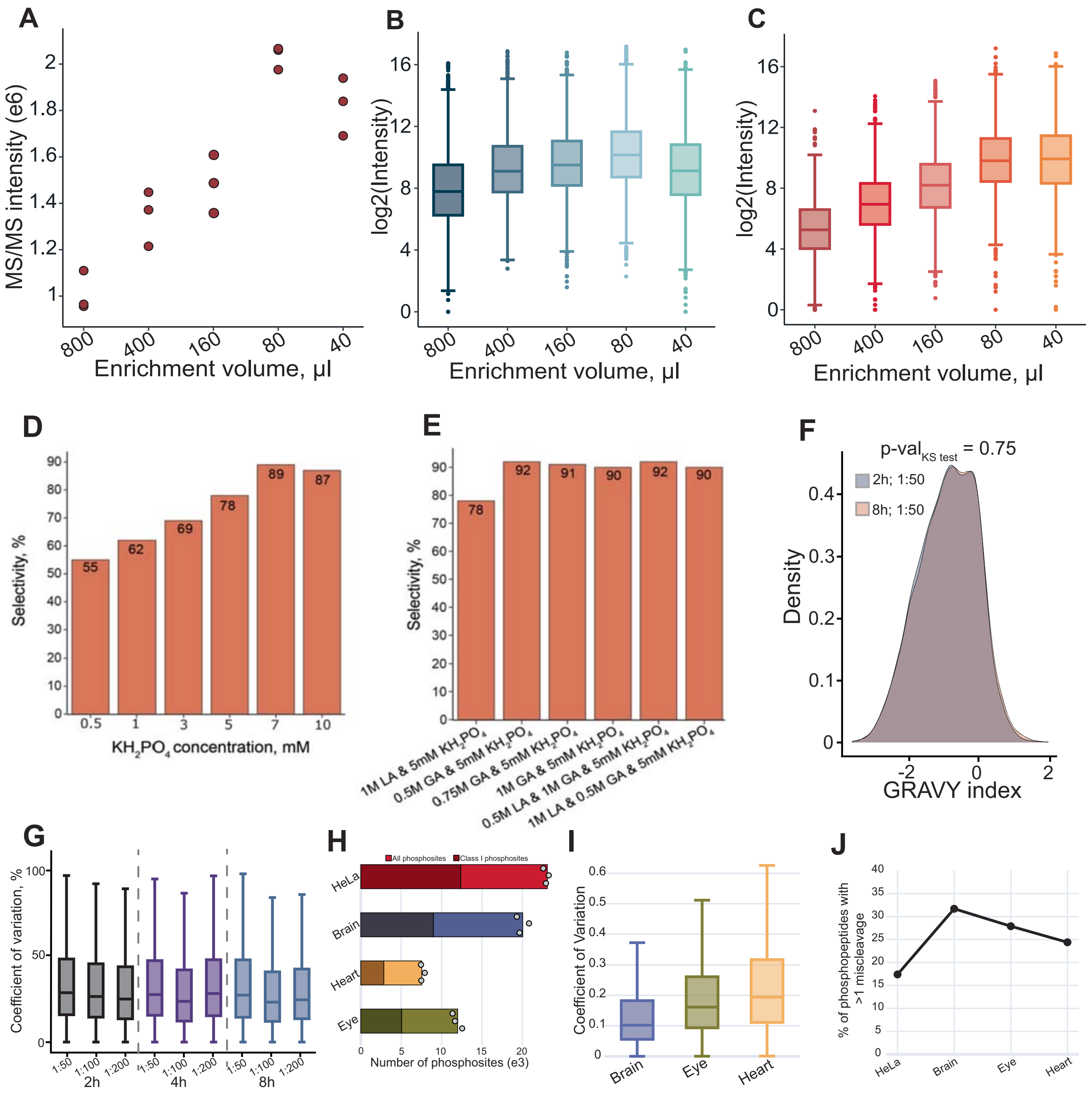

Figure EV2. Systematic optimization of experimental parameters for µPhos.

(**A**) Median raw fragment ion intensity in three replicate injections as a function of enrichment volume. (**B**) Logarithmized intensity of phosphopeptides for phosphopeptide enrichments with decreasing volume ($n = 3$). The box depicts the interquartile range with the central band representing the median value of the dataset. The whiskers represent the furthest datapoint within 1.5 times the interquartile range. (**C**) Same as (**B**), but for unmodified peptides ($n = 3$). The box depicts the interquartile range with the central band representing the median value of the dataset. The whiskers represent the furthest datapoint within 1.5 times the interquartile range. (**D**) Selectivity of phosphoenrichment as a function of increased concentration of monopotassium phosphate ($n = 1$). (**E**) Same as (**D**) but for combination of selectivity agents ($n = 1$). (**F**) Overlay of the GRAVY hydrophobicity index of detected phosphopeptides after either 2 h or 8 h digestion ($n = 3$). The significance of distribution overlay was determined using the Kolmogorov–Smirnov test. (**G**) Precision of label-free phosphopeptide quantification in workflow replicates ($n = 3$) for the conditions in Fig. 2D. The box depicts the interquartile range with the central band representing the median value of the dataset. The whiskers represent the furthest datapoint within 1.5 times the interquartile range. (**H**) Number of identified unique phosphosites (light colors) and Class 1 phosphosites (darker colors), enriched from 20 µg of mouse brain, heart, and eye tissue lysates and HeLa cell lysate ($n = 3$). (**I**) Precision of label-free phosphopeptide quantification in workflow replicates for the tissues in (**H**) ($n = 3$). The box depicts the interquartile range with the central band representing the median value of the dataset. The whiskers represent the furthest datapoint within 1.5 times the interquartile range. (**J**) Percent of phosphopeptides with ≥1 missed cleavage site for samples in (**H**).

© The Author(s)

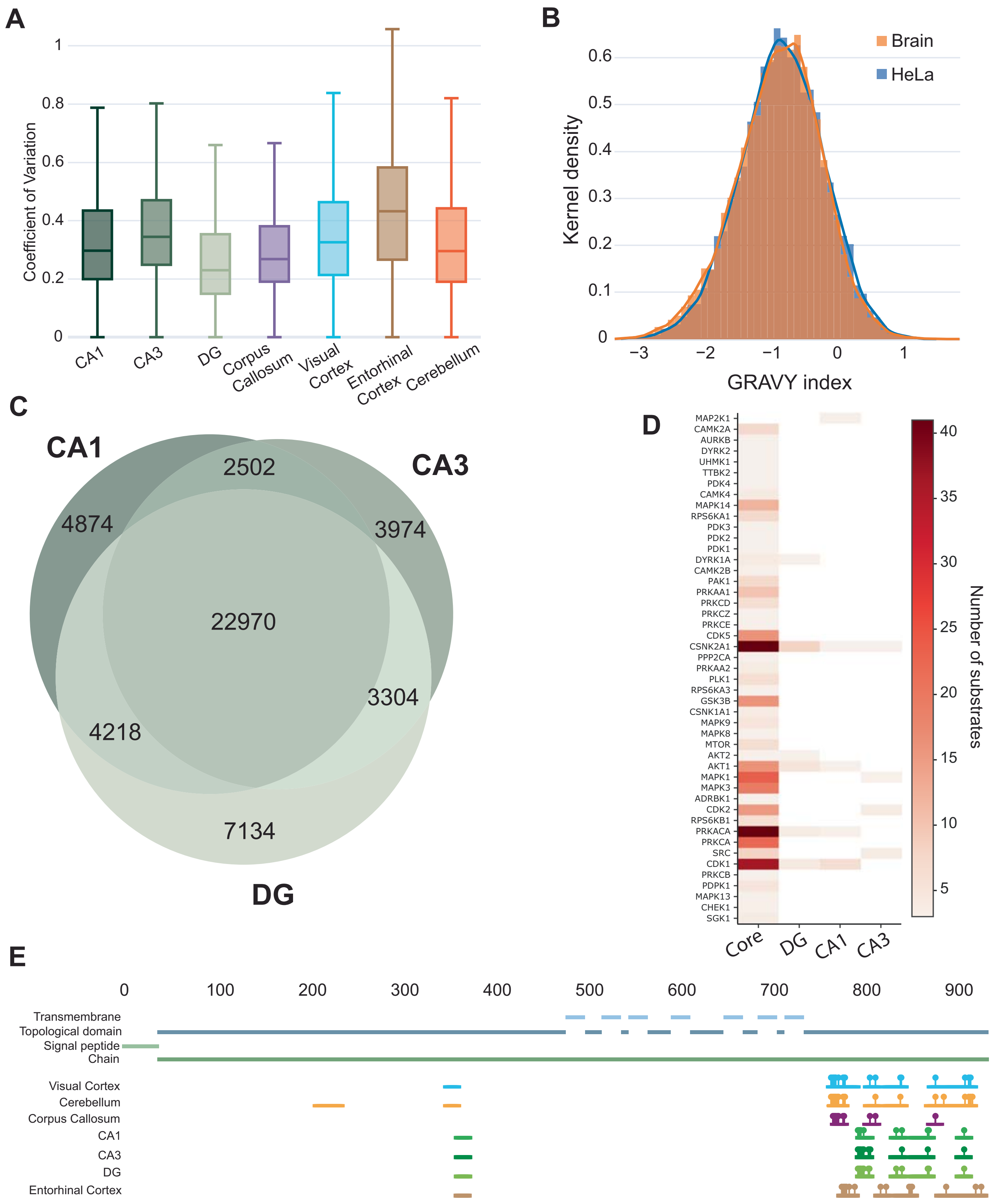
A
Coefficient of Variation
CA1
CA3
DG
Corpus Callosum
Visual Cortex
Entorhinal Cortex
Cerebellum
B
Kernel density
GRAVY index
Brain
HeLa
C
CA1
CA3
DG
2502
4874
3974
22970
3304
4218
7134
D
Number of substrates
Core
DG
CA1
CA3
E
Transmembrane
Topological domain
Signal peptide
Chain
Visual Cortex
Cerebellum
Corpus Callosum
CA1
CA3
DG
Entorhinal Cortex

 © The Author(s)

Figure EV3. Analysis of phosphoproteomes of anatomical regions in the mouse brain.

(**A**) Intrareplicate quantification precision of all analyzed mouse brain regions ($n = 3$). The box depicts the interquartile range with the central band representing the median value of the dataset. The whiskers represent the furthest datapoint within 1.5 times the interquartile range. (**B**) Overlay of the GRAVY hydrophobicity index of phosphopeptides, detected in mouse brain and HeLa samples. (**C**) Overlapping phosphopeptide identifications from subregions of the murine hippocampus. (**D**) Number of known substrates for selected kinases in overlapping ('core') and 'subregion-specific' phosphosites from (**C**). (**E**) AlphaMap(Voytik et al, 2022) visualization of identified phosphorylation sites on the Gabbr2 receptor in different samples.

© The Author(s)

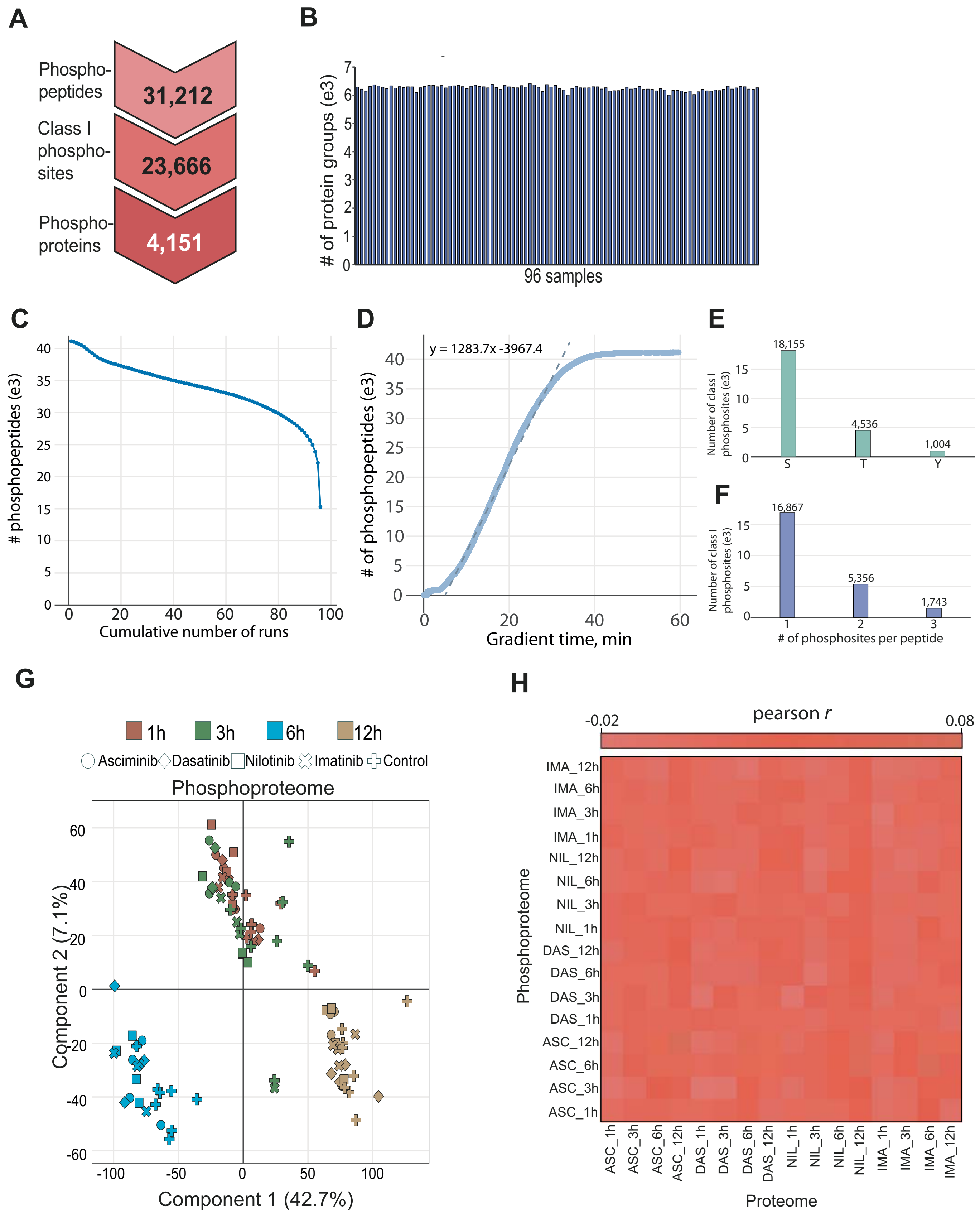
A
Phospho-peptides
31,212
Class I phospho-sites
23,666
Phospho-proteins
4,151
B
# of protein groups (e3)
96 samples
C
phosphopeptides (e3)
Cumulative number of runs
D
y = 1283.7x -3967.4
# of phosphopeptides (e3)
Gradient time, min
E
Number of class I phosphosites (e3)
18,155
4,536
1,004
S
T
Y
F
Number of class I phosphosites (e3)
16,867
5,356
1,743
# of phosphosites per peptide
G
1h
3h
6h
12h
Asciminib
Dasatinib
Nilotinib
Imatinib
Control
Phosphoproteome
Component 2 (7.1%)
Component 1 (42.7%)
H
pearson r
-0.02
0.08
IMA_12h
IMA_6h
IMA_3h
IMA_1h
NIL_12h
NIL_6h
NIL_3h
NIL_1h
DAS_12h
DAS_6h
DAS_3h
DAS_1h
ASC_12h
ASC_6h
ASC_3h
ASC_1h
Phosphoproteome
ASC_1h
ASC_3h
ASC_6h
ASC_12h
DAS_1h
DAS_3h
DAS_6h
DAS_12h
NIL_1h
NIL_3h
NIL_6h
NIL_12h
IMA_1h
IMA_3h
IMA_6h
IMA_12h
Proteome

 © The Author(s)

◀ **Figure EV4. Extended analysis of drug response signatures with μPhos.**

(**A**) Number of identified phosphosites, class 1 phosphosites and phosphorylated protein groups. (**B**) Number of identified protein groups across 96 samples. (**C**) Cumulative number of phosphopeptides identified in *n* out of 96 experiments. (**D**) Number of identified phosphopeptides as a function of the chromatographic gradient. For illustration, the slope in the active part of the gradient is determined by linear regression. (**E**) Relative number of phosphorylated serine, threonine, and tyrosine sites in our data. (**F**) Relative number of singly, doubly, and triply-phosphorylated peptides in our data. (**G**) Principal component analysis of the phosphoproteome samples. (**H**) Pairwise Pearson correlation analysis of fold-changes in protein and phosphoprotein levels relative to controls.

© The Author(s)

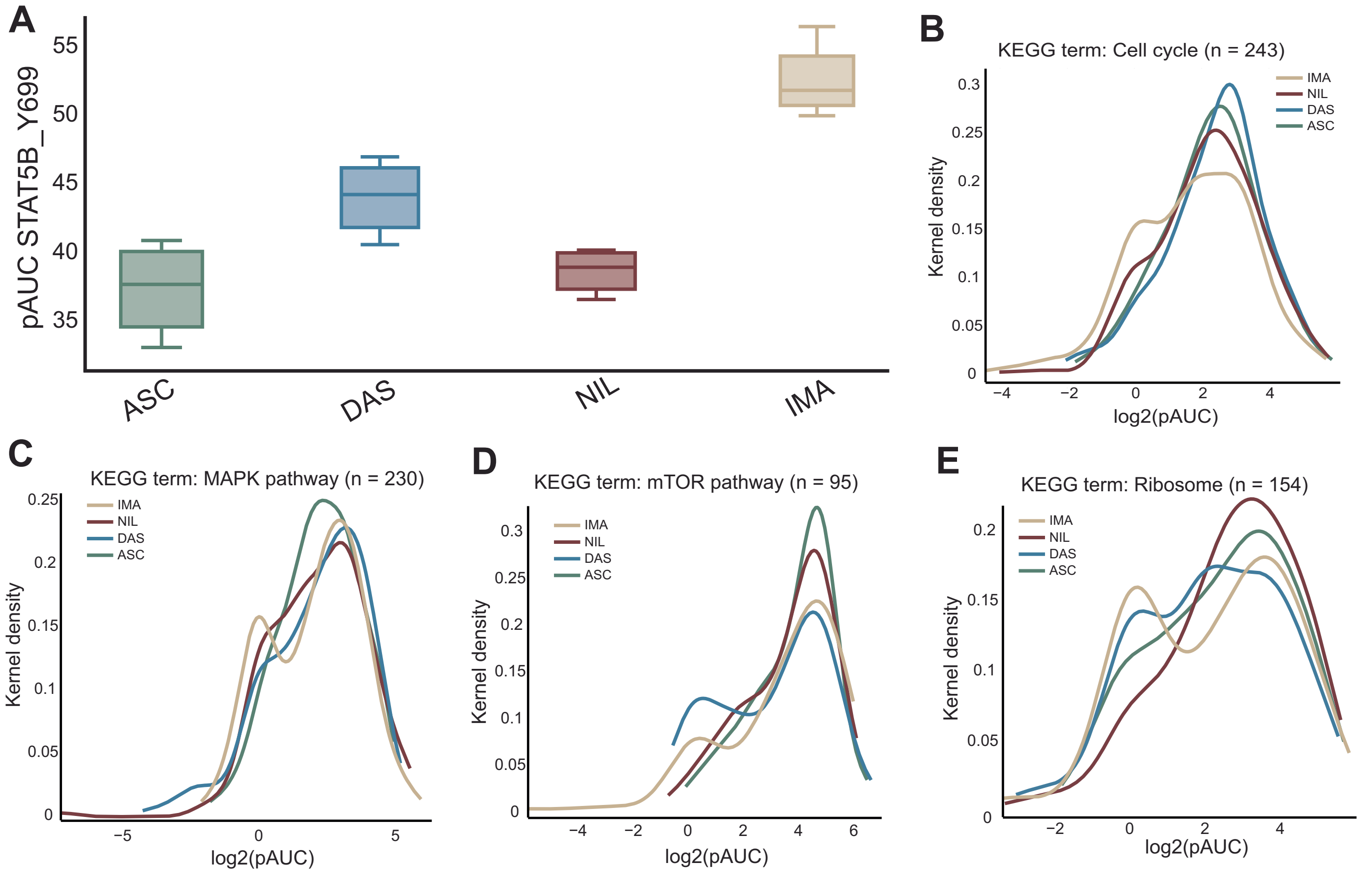

Figure EV5. Analysis of pAUC values for selected phosphorylation sites and signaling pathways.

(**A**) pAUC values of STAT5B Y699 in response to tyrosine kinase inhibition with different drugs ($n = 4$). The box depicts the interquartile range with the central band representing the median value of the dataset. The whiskers represent the furthest datapoint within 1.5 times the interquartile range. (**B**) Density distribution of pAUC values for phosphosites associated with the KEGG term "cell cycle". (**C**) Same as (**B**), but for the KEGG term 'MAPK pathway'. (**D**) Same as (**B**), but for the KEGG term 'mTOR pathway'. (**E**) Same as (**B**), but for the KEGG term 'Ribosome'.

 © The Author(s)
